# Supplementary figures and images for: hnRNP Q/SYNCRIP interacts with LIN28B and modulates the LIN28B/let-7 axis in human hepatoma cells
Source: PLoS One. 2024 Jul 8;19(7):e0304947. doi: 10.1371/journal.pone.0304947 (PMC11230530; doi:10.1371/journal.pone.0304947)

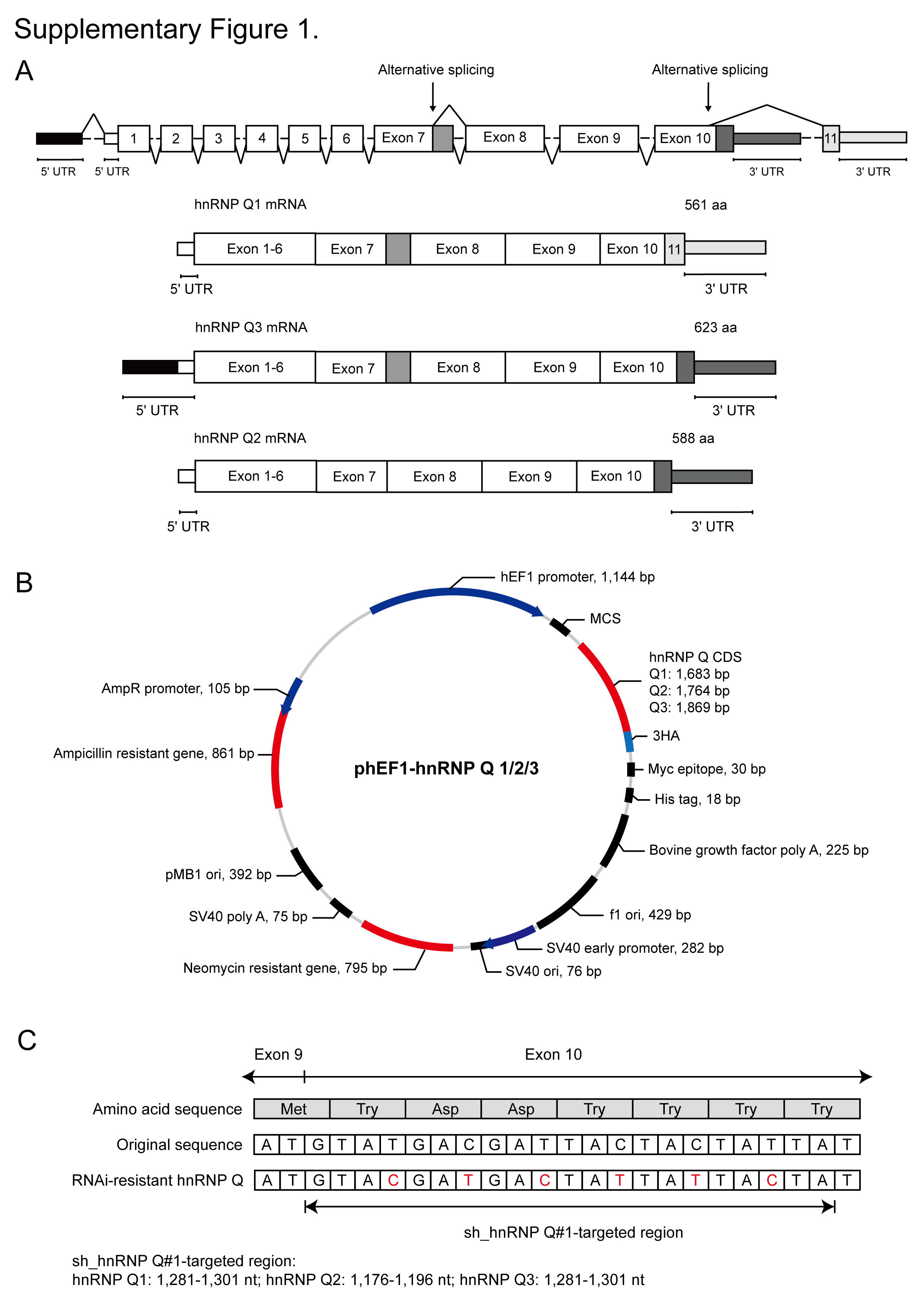

Supplement: S1 Fig — (A) Graphic depiction of the hnRNP Q gene. Three major isoforms are caused by alternative splicing. The longest isoform hnRNP Q3 represents most of the RGG box at the C-terminal. hnRNP Q2 contains a truncated second RBD due to alternative splicing at exon 7. hnRNP Q1 contains a truncated C-terminal due to alternative splicing. (B) Graphical depiction of the pEF-hnRNP Q1/2/3 constructs. (C) Design of the RNAi-resistant sequence for hnRNP Q. (TIF) [file pone.0304947.s001.tif]

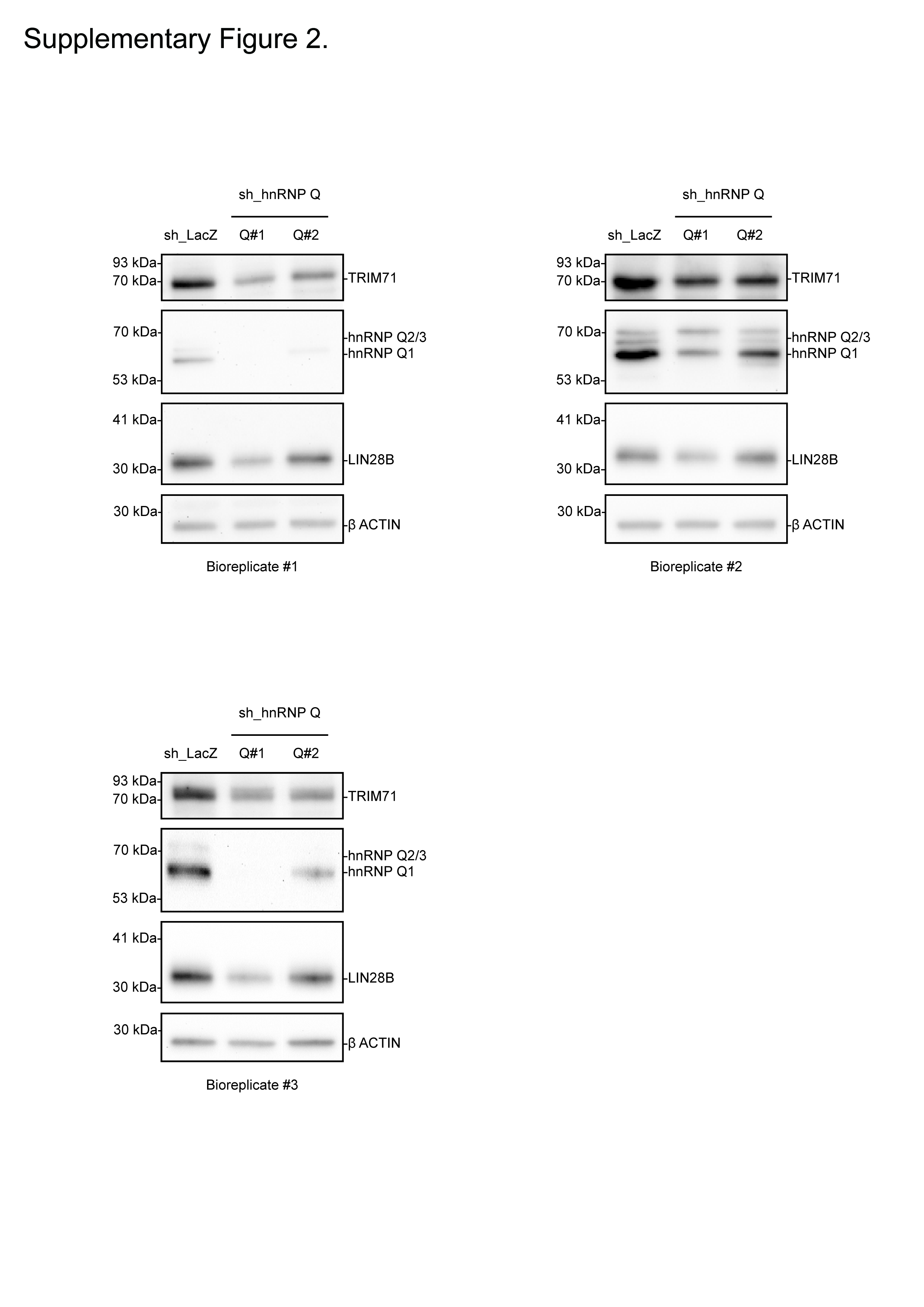

Supplement: S2 Fig — Three other biological replicates that accompany Fig 2A. The protein levels of hnRNP Q, TRIM71 and LIN28 were analyzed by western blot. Actin served as an internal control. (TIF) [file pone.0304947.s002.tif]

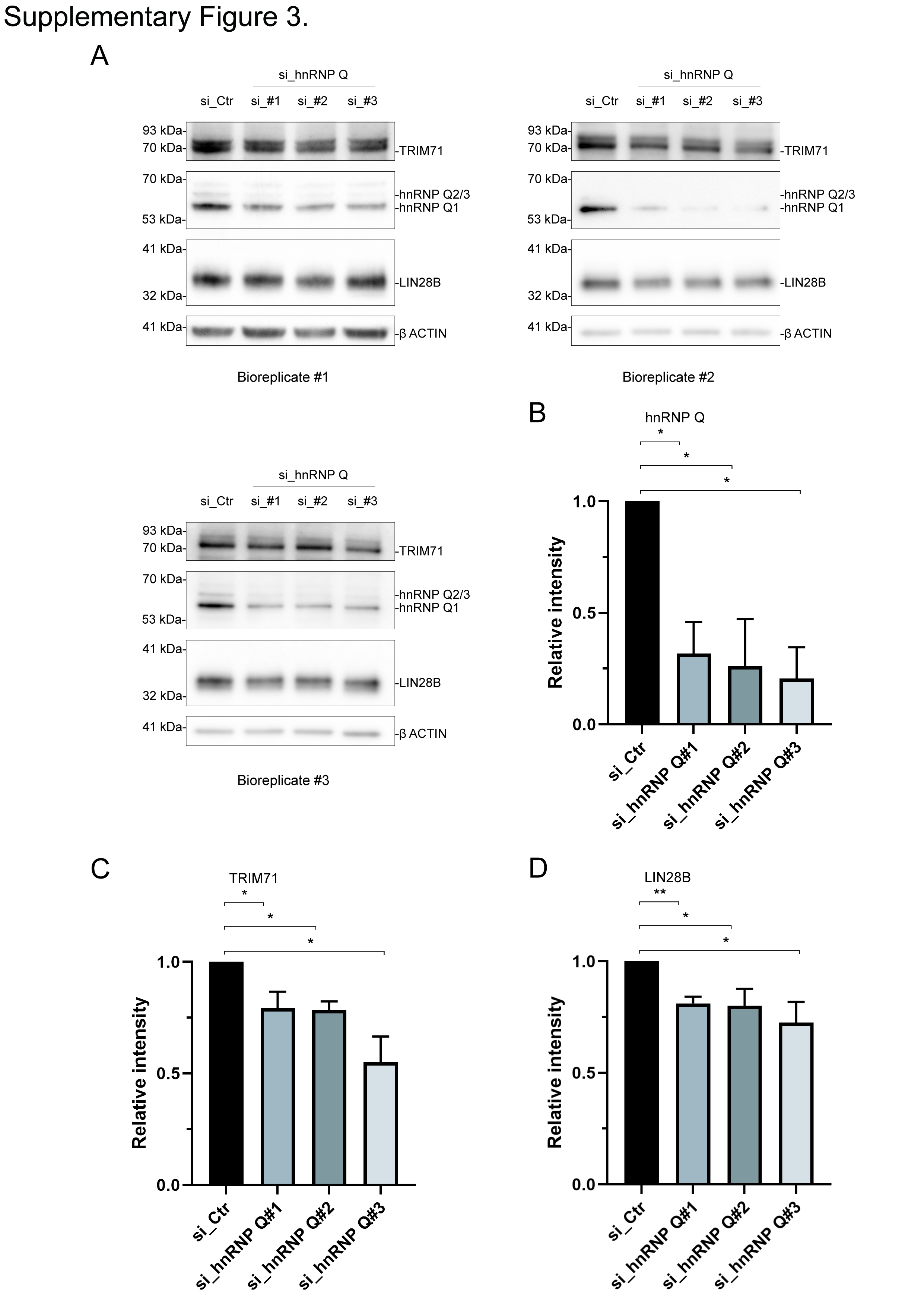

Supplement: S3 Fig — Huh7 cells were transfected with three different siRNAs against hnRNP Q. The protein level of hnRNP Q, TRIM71 and LIN28 were analyzed by western blot. Actin served as an internal control. (A) Two other biological replicates for hnRNP Q depletion by shRNAs reduces protein levels of TRIM71 and LIN28B in Huh7 cells. (B) Quantification of hnRNP Q levels in Huh7 cells with siRNA. (C) Quantification of TRIM71 levels in hnRNP Q depleted Huh7 cells. (D) Quantification of LIN28B levels in hnRNP Q depleted Huh7 cells. Results are plotted as average ± S.D., *P < 0.05, **P < 0.01, ***P < 0.001 using an unpaired two-tailed Student’s t-test. (TIF) [file pone.0304947.s003.tif]

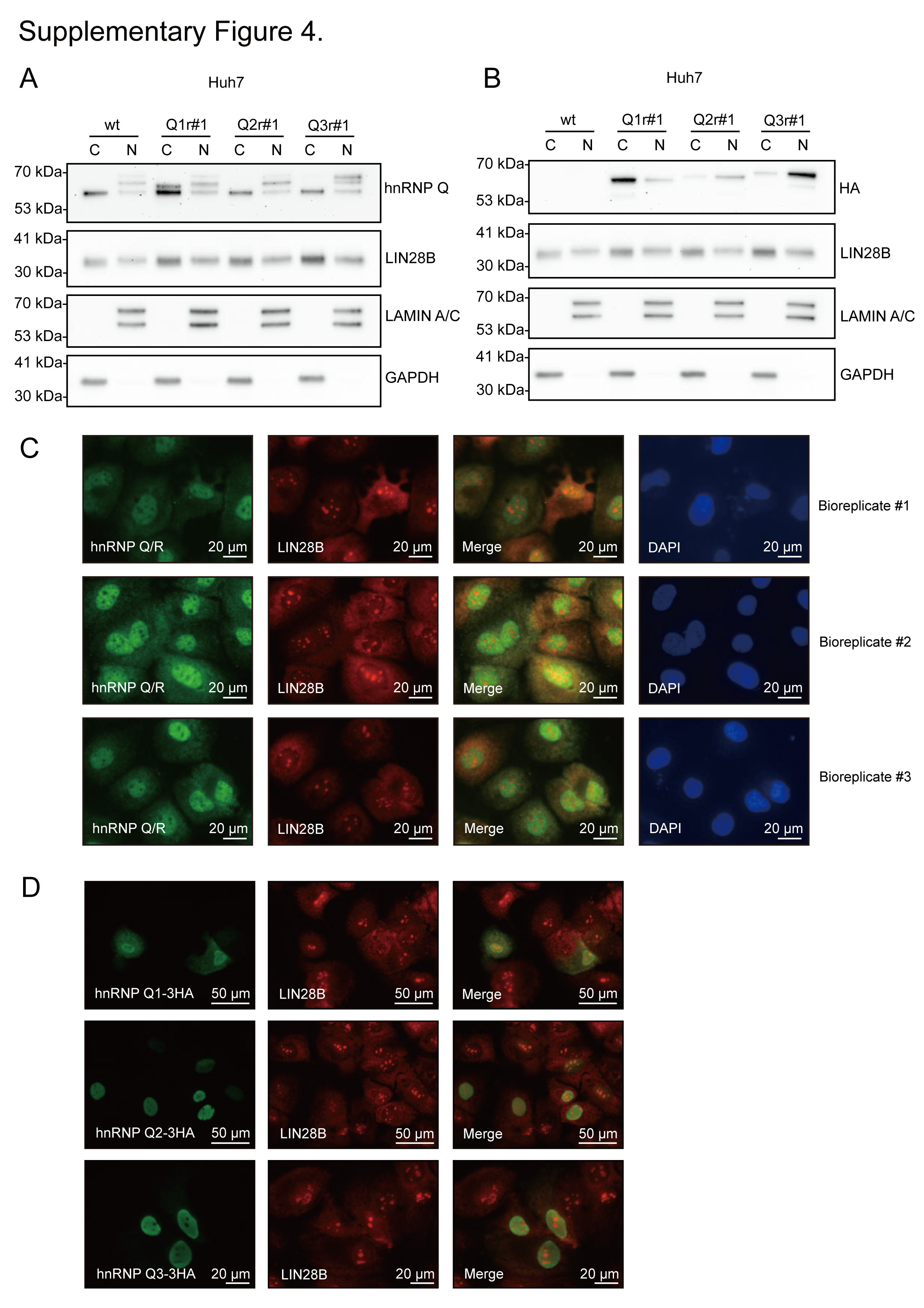

Supplement: S4 Fig — (A) Nuclear/cytoplasmic fractionation of Huh7 cells without or with transfected HA-tagged hnRNP Q isoforms. The distribution of hnRNP Q isoforms for both the wild-type hnRNP Q and HA-tagged hnRNP Q was detected using an anti-hnRNP Q antibody. hnRNP Q1 predominantly localized to the cytoplasm while the vast majority of hnRNP Q2 and 3 was found in the nucleus. We found that LIN28B was detected in both the nucleus and cytoplasm. LAMIN A/C and GAPDH served as nuclear and cytoplasmic markers, respectively (B) The HA-tagged hnRNP Q isoforms were detected by western blot using an anti-HA antibody. The localization of HA-tagged hnRNP Q isoforms was the same as observed with endogenous proteins. (C) Immunocytochemistry of endogenous hnRNP Q and LIN28B in Huh7 cells. hnRNP Q (labeled in green) was found in both the cytoplasm and the nucleus while LIN28B (labeled in red) localized to the cytoplasm and the nucleolus. DAPI staining indicated the nucleus. Three biological replicates are shown. (D) Immunocytochemistry of HA-tagged hnRNP Q isoforms and endogenous Lin28B in Huh7 cells. HA-tagged hnRNP Q1 localized to the cytoplasm and the nucleus while hnRNP Q2 and 3 predominantly localized to the nucleus. (TIF) [file pone.0304947.s004.tif]

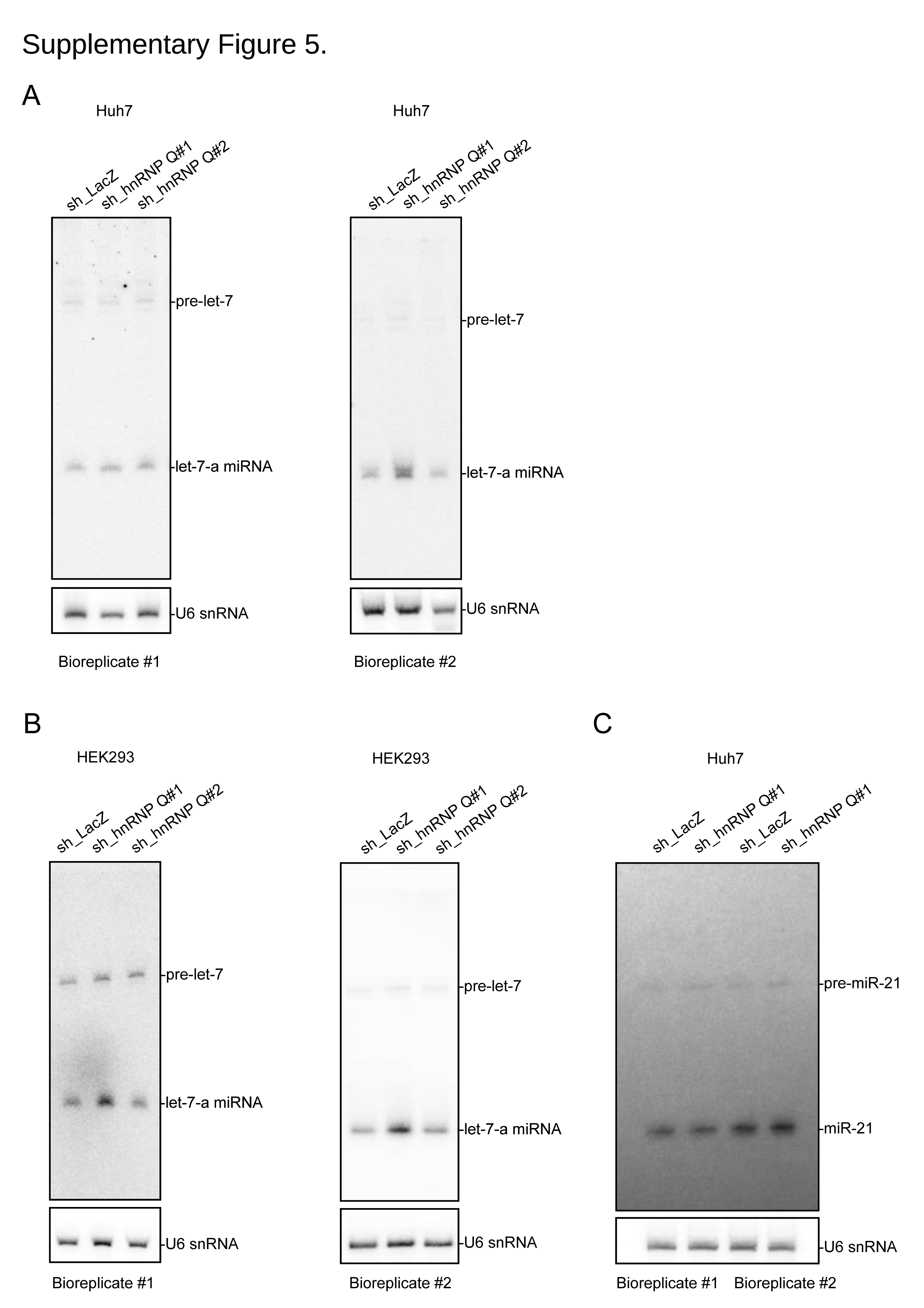

Supplement: S5 Fig — (TIF) [file pone.0304947.s005.tif]

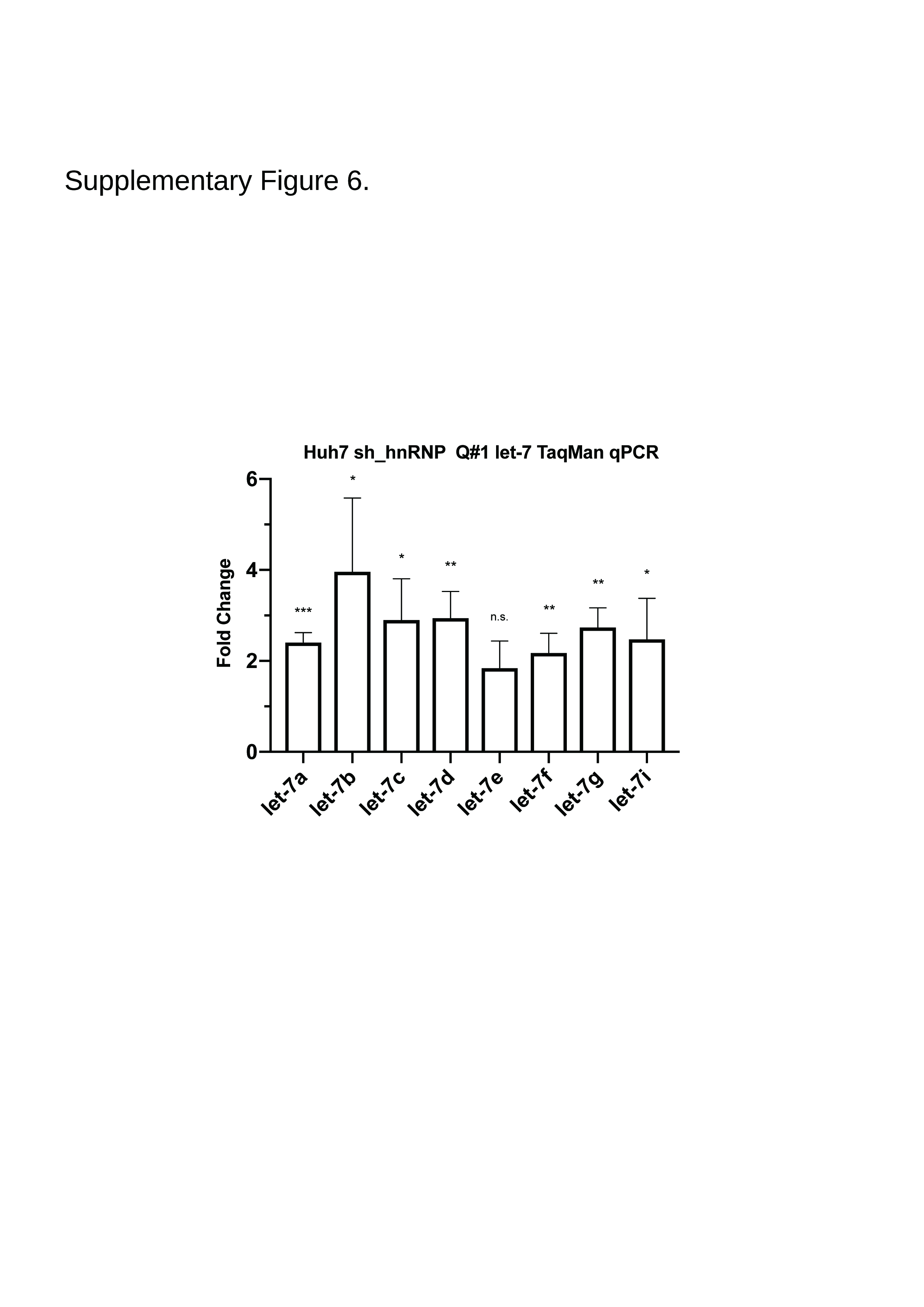

Supplement: S6 Fig — Data were shown as foldchange (Ctr = 1) ± S.D. from three independent experiments, *P < 0.05, **P < 0.01, ***P < 0.001 using an unpaired two-tailed Student’s t-test. RNU48 snoRNA was used as an endogenous control. (TIF) [file pone.0304947.s006.tif]

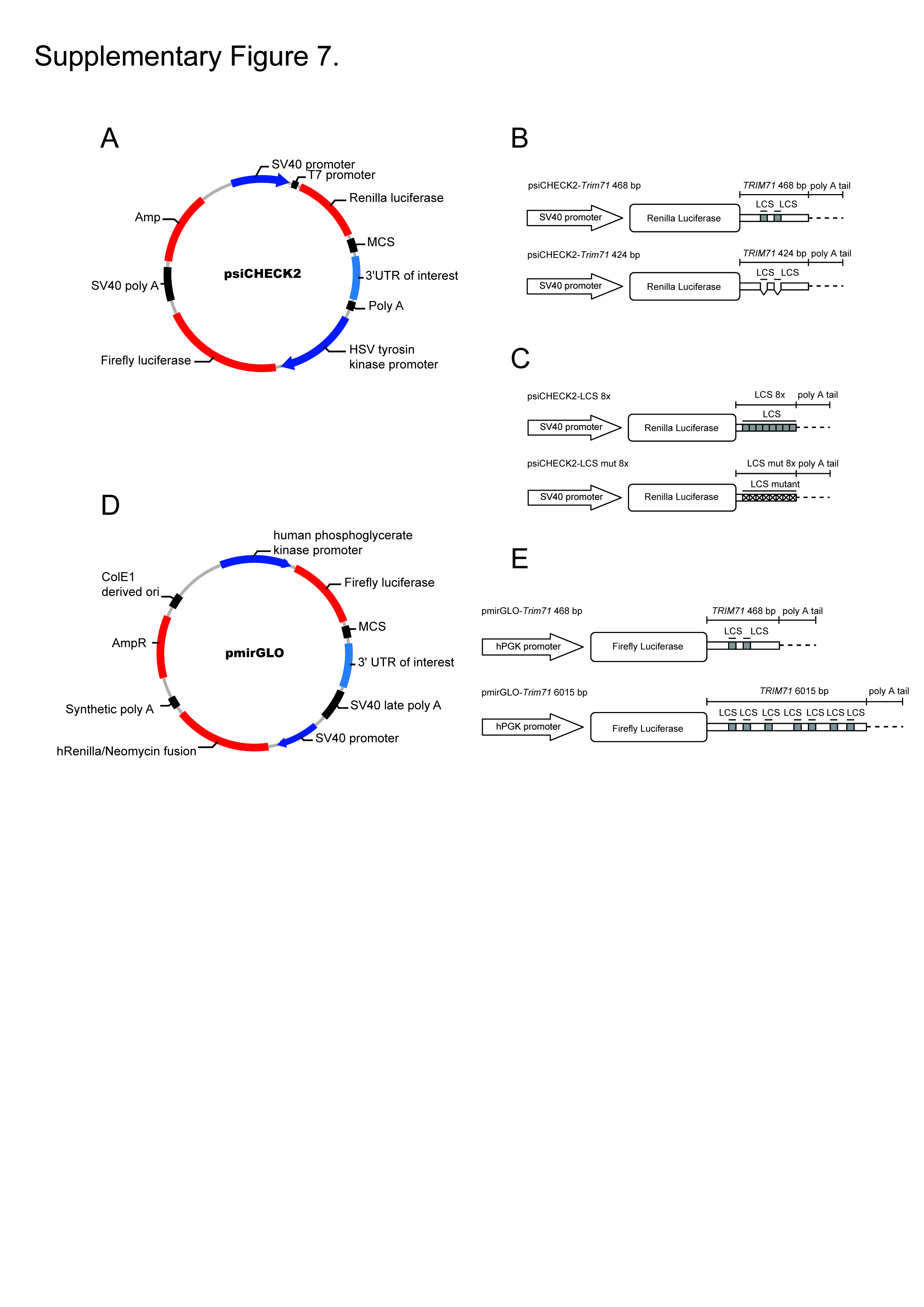

Supplement: S7 Fig — (A) The psiCHECK2 vector. (B) A 468-bp TRIM71 3’UTR and a 424-bp TRIM71 3’ UTR with two LCSs deleted were cloned into the psiCHECK2 downstream of the renilla luciferase gene. (C) 3’UTR sequences containing eight copies of LCSs or mutated LCSs were cloned into the psiCHECK2 vector. (D) The pmirGLO vector. (E) A 468-bp TRIM71 3’UTR and a 6015-bp TRIM71 3’ UTR were cloned into the pmirGLO after the firefly luciferase gene. (TIF) [file pone.0304947.s007.tif]
