# Supplementary material for: hnRNP Q/SYNCRIP interacts with LIN28B and modulates the LIN28B/let-7 axis in human hepatoma cells
Source: PLoS One. 2024 Jul 8;19(7):e0304947. doi: 10.1371/journal.pone.0304947 (PMC11230530; doi:10.1371/journal.pone.0304947)
Supplement: S1 Table — (PDF) [file pone.0304947.s008.pdf]

Supplementary Table 1. The primers used in this work.

Name	Sequences
**For phEF1-hnRNP Q3HA cloning[1]**	
HA Fwd	5' ACAAGCGGCCGCATCTAGAATGTACCCATACGATGTTCCAG 3'
HA Rev	5' TTTGTTCGAATCAGAATTCGGATCCAGCGTAATCTGG 3'
**For RNAi-resistant hnRNP Q cloning[2]**	
hnRNP Qrsh_Q#1 Fwd1	5' GATCCACTAGTCCAGTGTGGTGGA 3'
hnRNP Qrsh_Q#1 Rev1	5' TAGTAATAATAGTCATCGTACATTTGATTTTTTGCTGCTTGCCTCTGAG 3'
hnRNP Qrsh_Q#1 Fwd2	5' GTACGATGACTATTATTACTATGGTCCACCTCATATGCCCCC 3'
hnRNP Qrsh_Q#1 Rev2	5' CAGATCCTCTTCTGAGATGAGTTTTTGTTC 3'
**For *Trim71* 3' UTR cloning[3]**	
Trim71 3' UTR Fwd1	5' GATATGCACAAGCCTGGCATCTGTATG 3'
Trim71 3' UTR Rev1	5' GAAAAGACATTACATCCTACATTTCAGGTTGGGAC 3'
Trim71 3' UTR Fwd2	5' GTCCCAACCTGAAATGTAGGATGTAATGTCTTTTC 3'
Trim71 3' UTR Rev2	5' CTGGCATTACCCGGGCAGGAGATTATGCATTTAAAC 3'
Trim71 3' UTR Fwd3	5' GTTTAAATGCATAATCTCCTGCCCGGGTAATGCCAG 3'
Trim71 3' UTR Rev3	5' CTGCCGGGTCCAGAGAGCTACAAATACAATC 3'

Trim71 3' UTR Fwd4	5' GATTGTATTTGTAGCTCTCTGGACCCGGCAG 3'
Trim71 3' UTR Rev4	5' GTTAAAAATTAAAGCATTTTGCTGACCCGGCATGAG 3'
Trim71 3' UTR Fwd5	5' CTCATGCCGGGTCAGCAAAATGCTTTAATTTTTAAC 3'
Trim71 3' UTR Rev5	5' GCGGCCAGCGGCCGCTAGCCATGGAAAAACCCTCGTTTATTTGATTAAAC 3'

[1]The cloning sites for *Bst*BI and *Not*I are indicated by the bottom lines.

[2]Mutated nucleotide positions for RNAi resistance are indicated by the bottom lines.

The construction of the extended 6015 bp TRIM71 3' UTR was achieved by multiple Gibson Assembly of five PCR fragments from the genomic DNA.
