# Supplementary material for: hnRNP Q/SYNCRIP interacts with LIN28B and modulates the LIN28B/let-7 axis in human hepatoma cells
Source: PLoS One. 2024 Jul 8;19(7):e0304947. doi: 10.1371/journal.pone.0304947 (PMC11230530; doi:10.1371/journal.pone.0304947)

Figure 1A

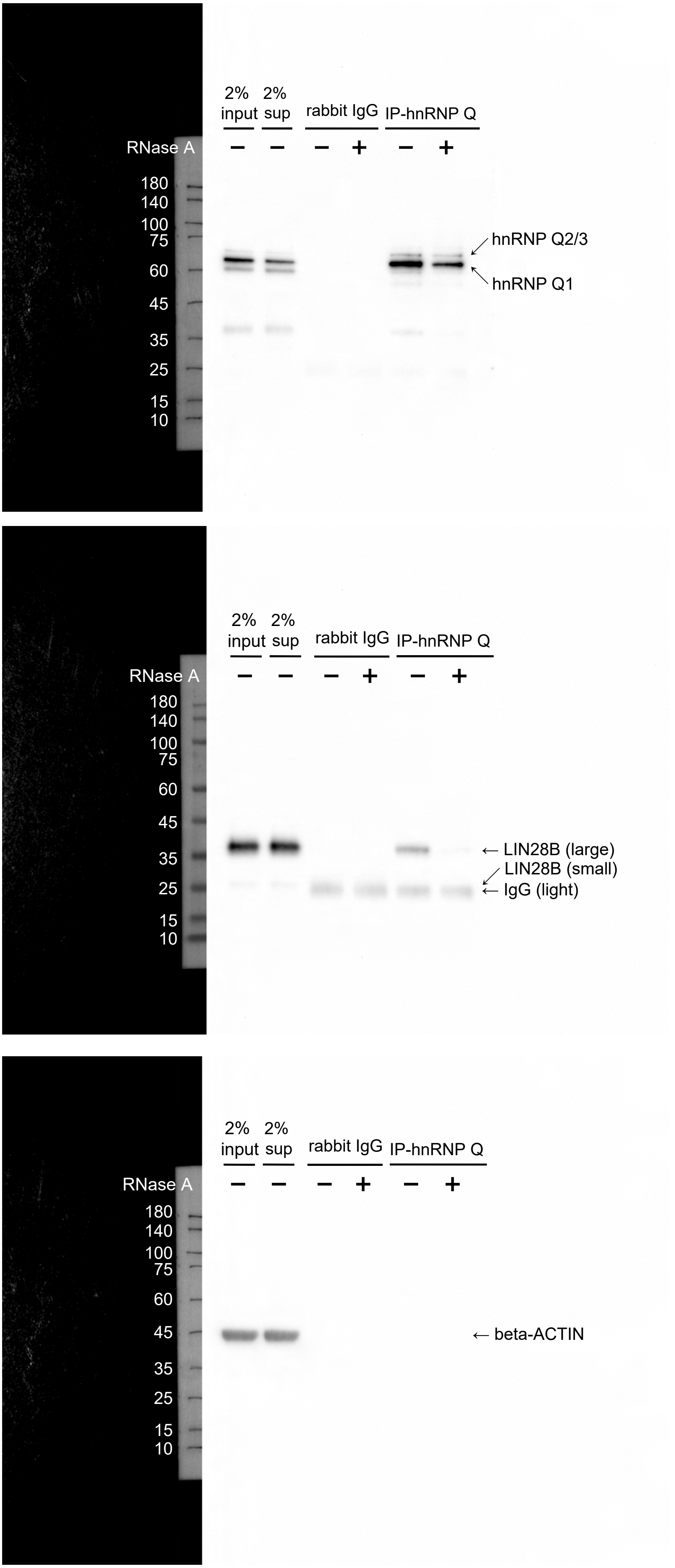

2% input
2% sup
rabbit IgG
IP-hnRNP Q
RNase A
180
140
100
75
60
45
35
25
15
10
hnRNP Q2/3
hnRNP Q1
2% input
2% sup
rabbit IgG
IP-hnRNP Q
RNase A
180
140
100
75
60
45
35
25
15
10
LIN28B (large)
LIN28B (small)
IgG (light)
2% input
2% sup
rabbit IgG
IP-hnRNP Q
RNase A
180
140
100
75
60
45
35
25
15
10
beta-ACTIN

Figure 1B

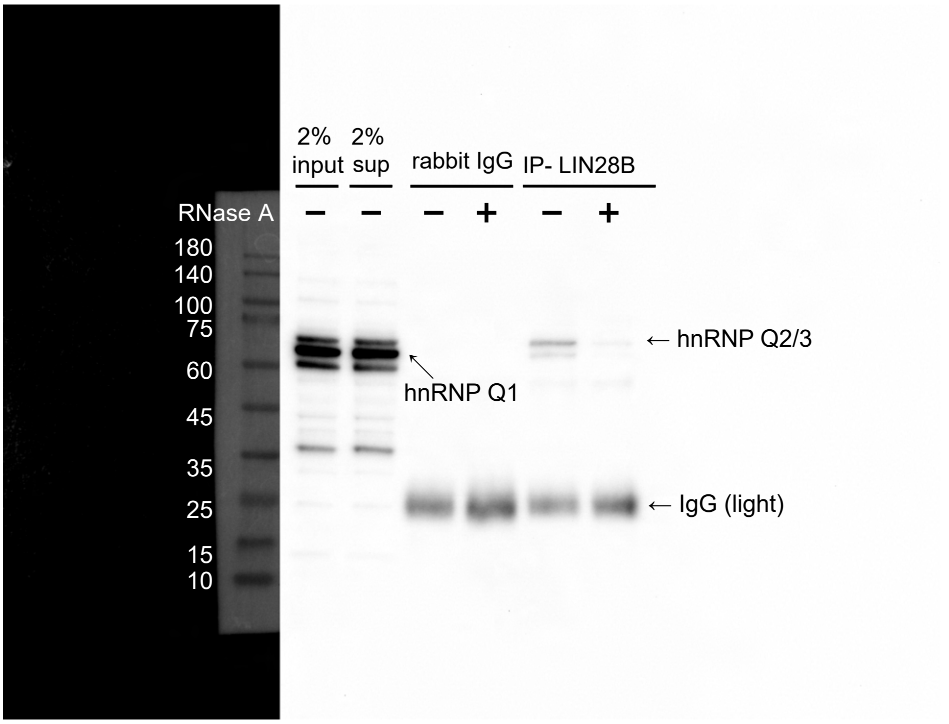
2% input
2% sup
rabbit IgG
IP- LIN28B
RNase A
− − − + − +
180
140
100
75
60
45
35
25
15
10
← hnRNP Q2/3
hnRNP Q1
← IgG (light)

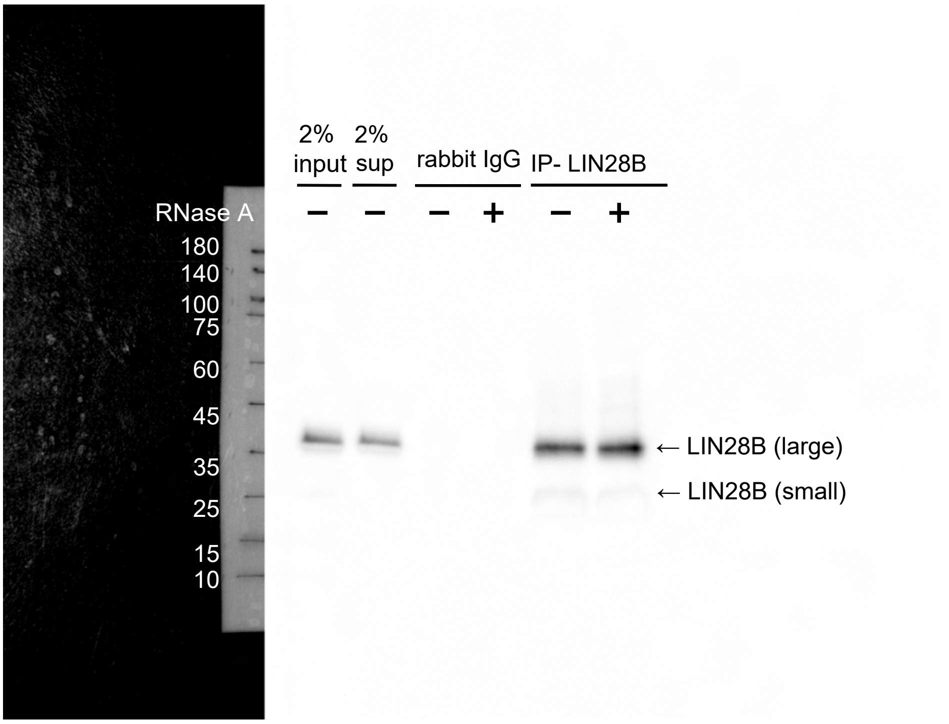
2% input
2% sup
rabbit IgG
IP- LIN28B
RNase A
− − − + − +
180
140
100
75
60
45
35
25
15
10
← LIN28B (large)
← LIN28B (small)

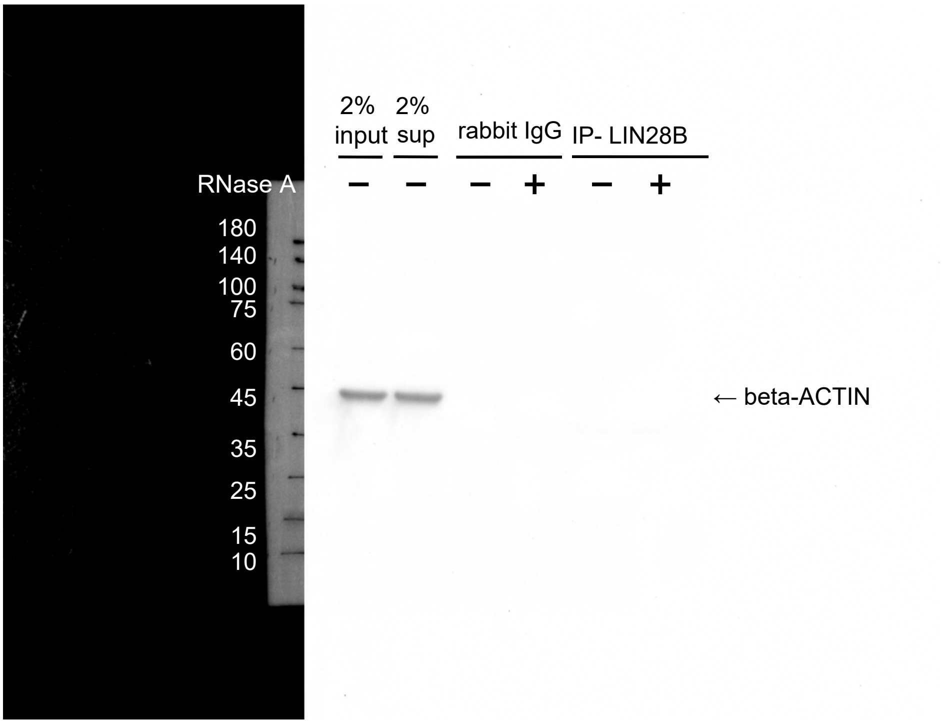
2% input
2% sup
rabbit IgG
IP- LIN28B
RNase A
− − − + − +
180
140
100
75
60
45
35
25
15
10
← beta-ACTIN

Figure 1C

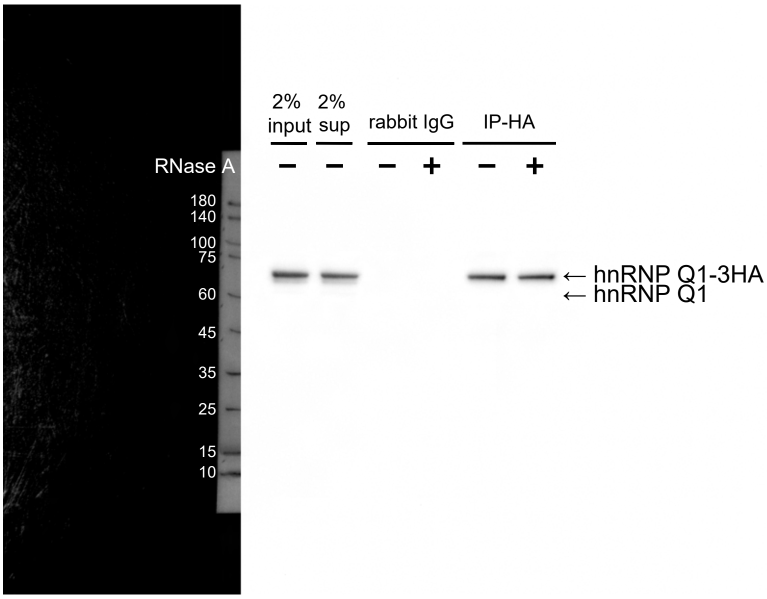
2% input
2% sup
rabbit IgG
IP-HA
RNase A
− − − + − +
180
140
100
75
60
45
35
25
15
10
← hnRNP Q1-3HA
← hnRNP Q1

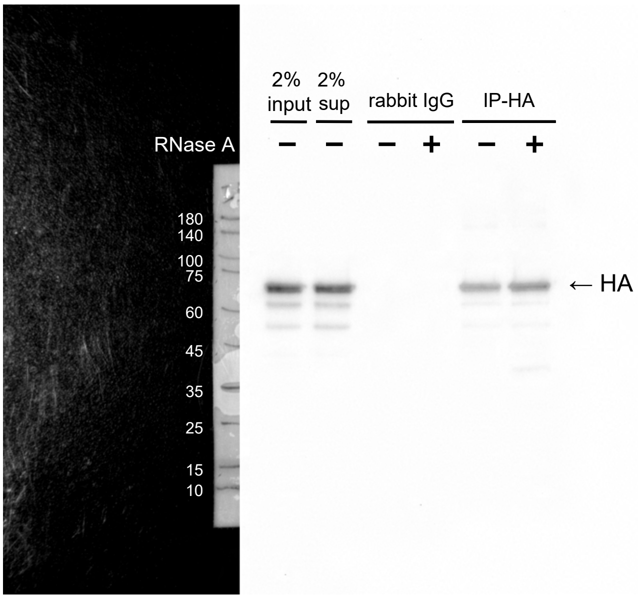
2% input
2% sup
rabbit IgG
IP-HA
RNase A
− − − + − +
180
140
100
75
60
45
35
25
15
10
← HA

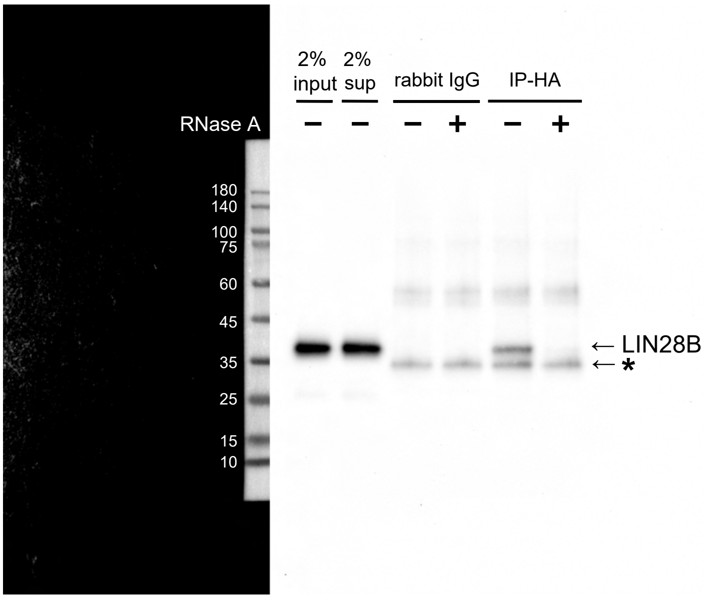
2% input
2% sup
rabbit IgG
IP-HA
RNase A
− − − + − +
180
140
100
75
60
45
35
25
15
10
← LIN28B
← *

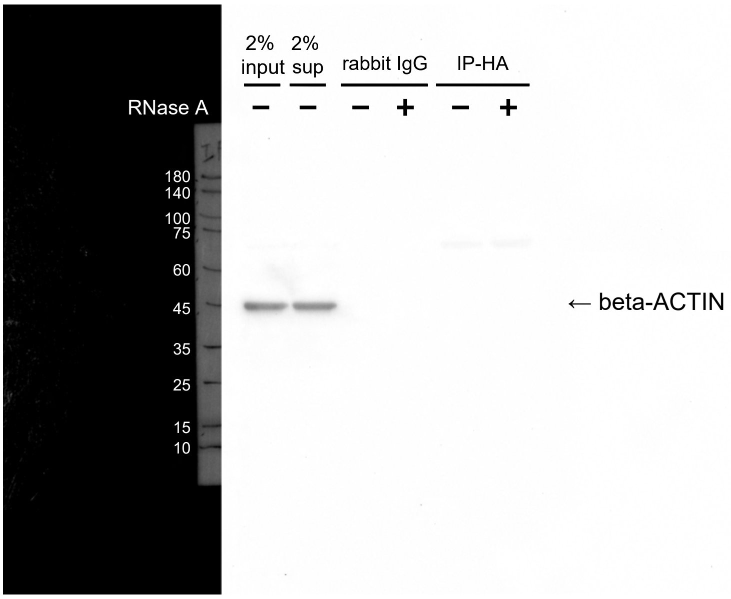
2% input
2% sup
rabbit IgG
IP-HA
RNase A
− − − + − +
180
140
100
75
60
45
35
25
15
10
← beta-ACTIN

Figure 1D

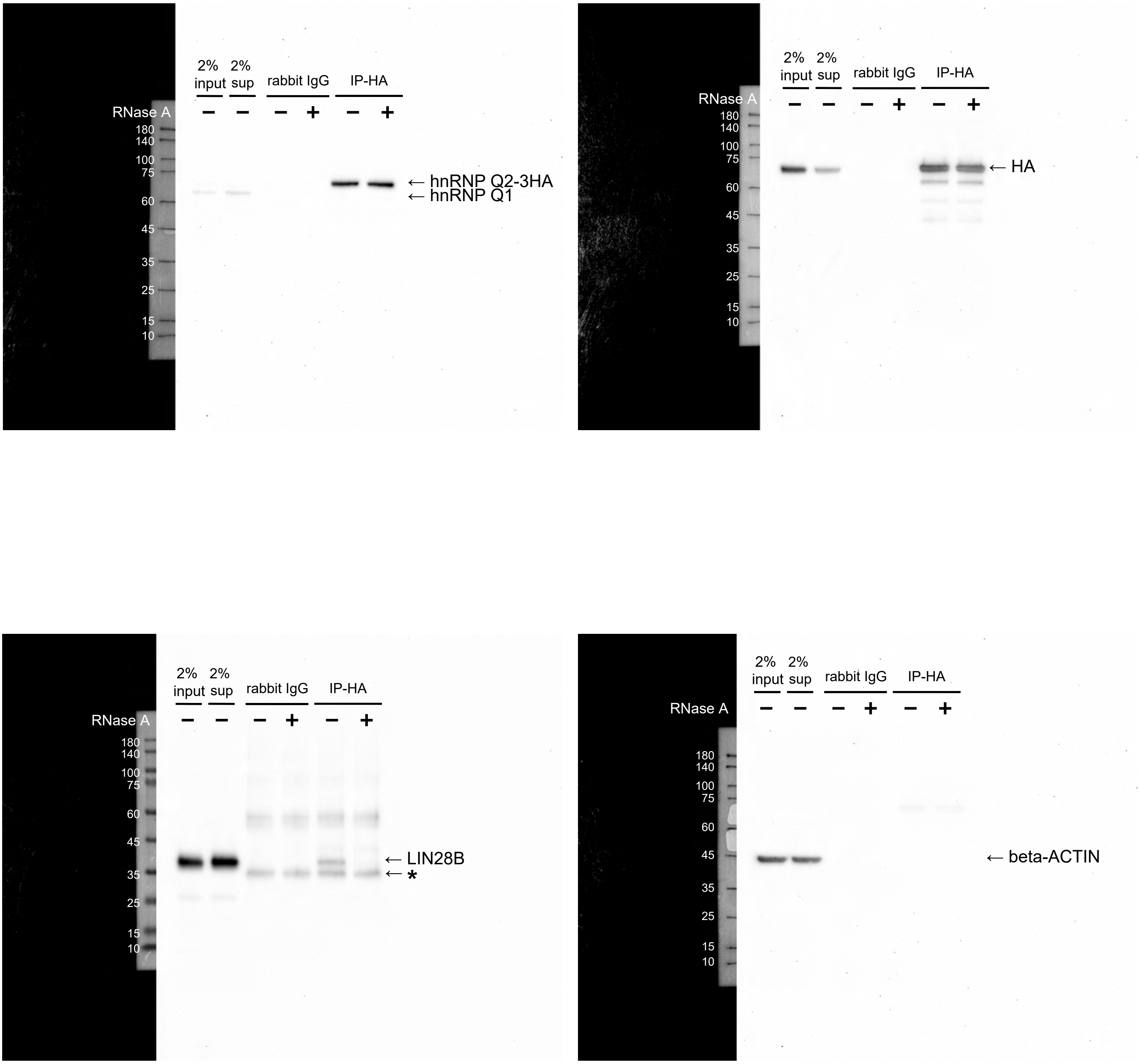
2% input
2% sup
rabbit IgG
IP-HA
RNase A
− − − + − +
180
140
100
75
60
45
35
25
15
10
← hnRNP Q2-3HA
← hnRNP Q1
2% input
2% sup
rabbit IgG
IP-HA
RNase A
− − − + − +
180
140
100
75
60
45
35
25
15
10
← HA
2% input
2% sup
rabbit IgG
IP-HA
RNase A
− − − + − +
180
140
100
75
60
45
35
25
15
10
← LIN28B
← *
2% input
2% sup
rabbit IgG
IP-HA
RNase A
− − − + − +
180
140
100
75
60
45
35
25
15
10
← beta-ACTIN

Figure 1E

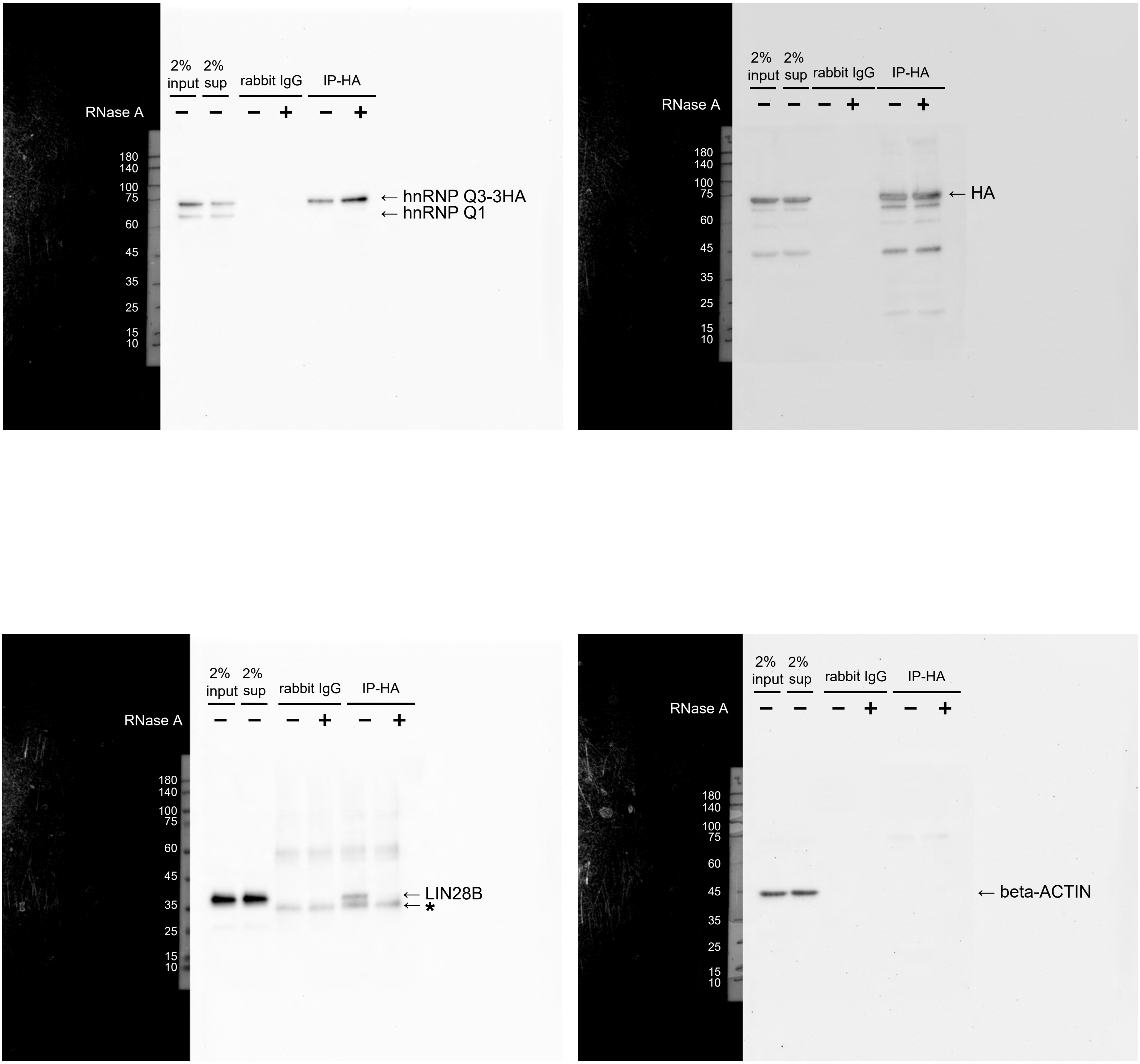
2% input
2% sup
rabbit IgG
IP-HA
RNase A
180
140
100
75
60
45
35
25
15
10
← hnRNP Q3-3HA
← hnRNP Q1
← HA
← LIN28B
← *
← beta-ACTIN

Figure 2A

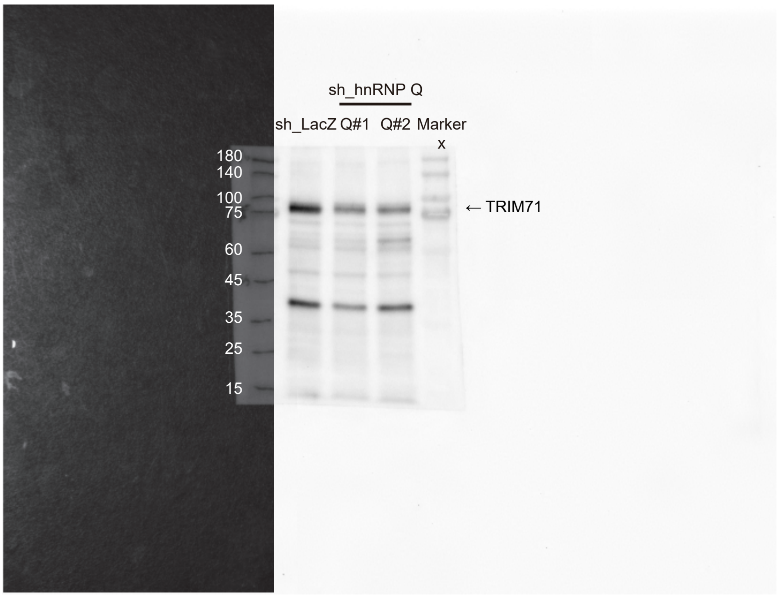
sh_hnRNP Q
sh_LacZ Q#1 Q#2 Marker
x
180
140
100
75
60
45
35
25
15
← TRIM71

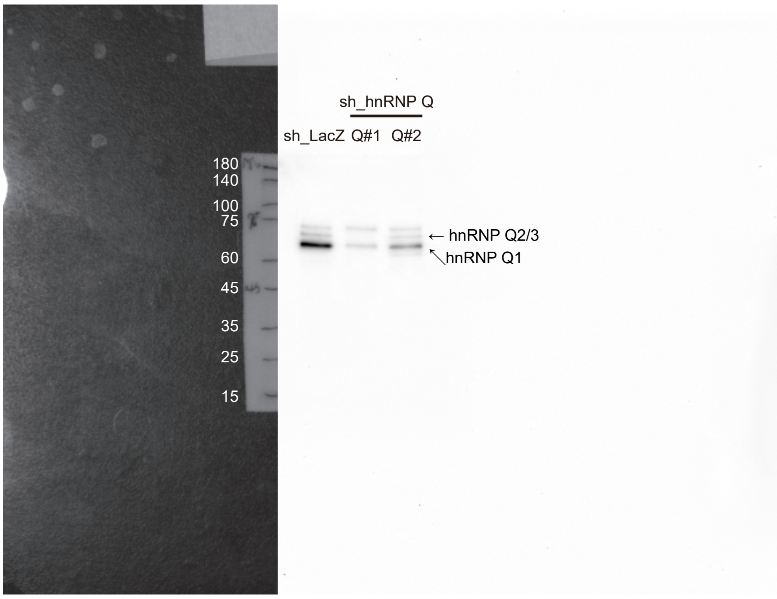
sh_hnRNP Q
sh_LacZ Q#1 Q#2
180
140
100
75
60
45
35
25
15
← hnRNP Q2/3
hnRNP Q1

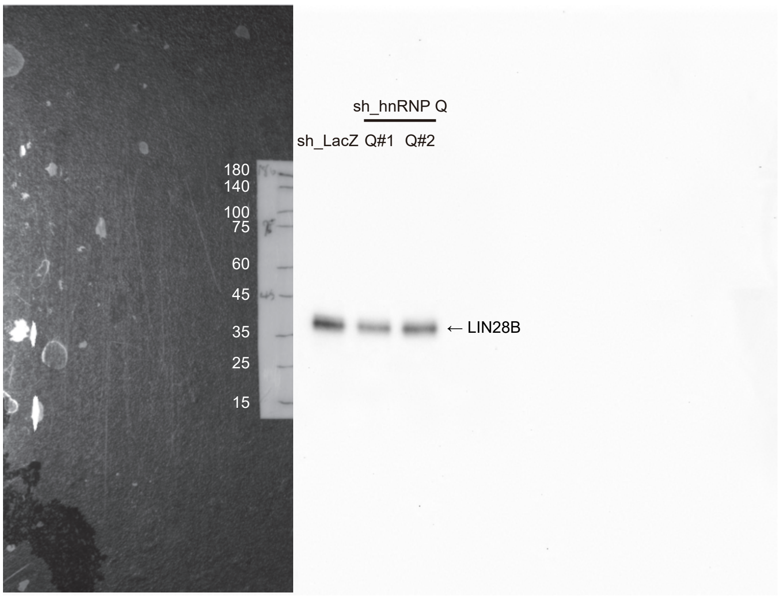
sh_hnRNP Q
sh_LacZ Q#1 Q#2
180
140
100
75
60
45
35
25
15
← LIN28B

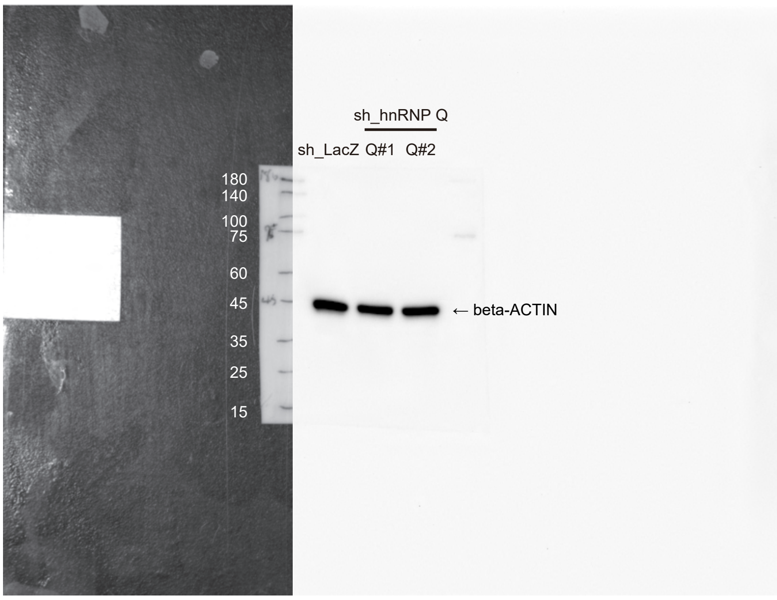
sh_hnRNP Q
sh_LacZ Q#1 Q#2
180
140
100
75
60
45
35
25
15
← beta-ACTIN

Figure 2E

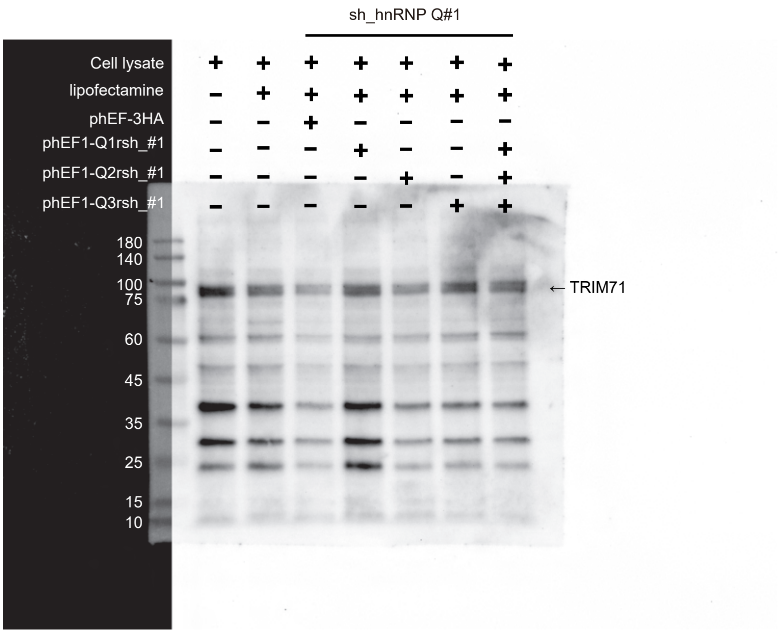
sh_hnRNP Q#1
Cell lysate
lipofectamine
phEF-3HA
phEF1-Q1rsh_#1
phEF1-Q2rsh_#1
phEF1-Q3rsh_#1
180
140
100
75
60
45
35
25
15
10
← TRIM71

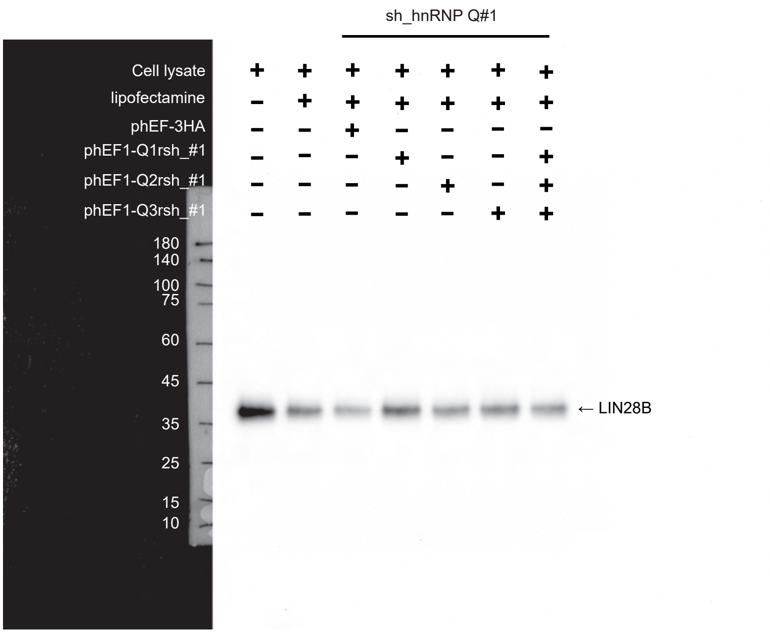
sh_hnRNP Q#1
Cell lysate
lipofectamine
phEF-3HA
phEF1-Q1rsh_#1
phEF1-Q2rsh_#1
phEF1-Q3rsh_#1
180
140
100
75
60
45
35
25
15
10
← LIN28B

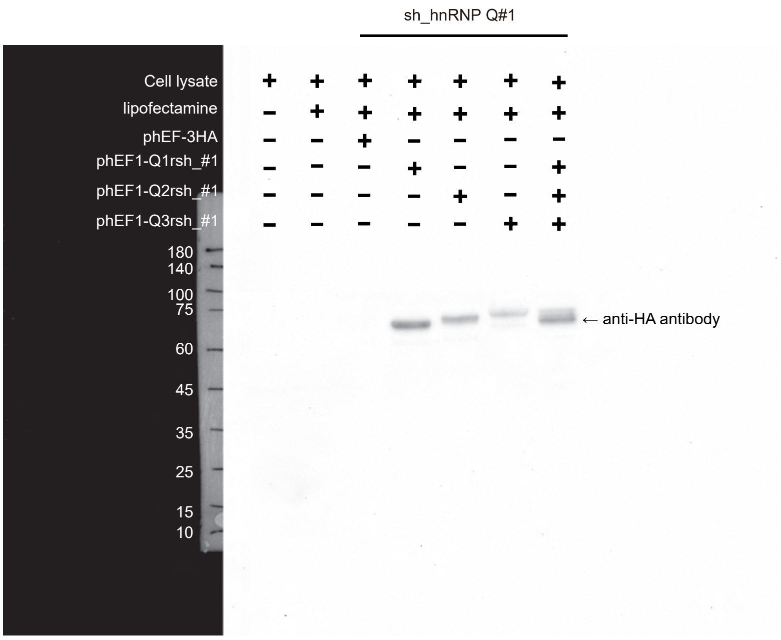
sh_hnRNP Q#1
Cell lysate
lipofectamine
phEF-3HA
phEF1-Q1rsh_#1
phEF1-Q2rsh_#1
phEF1-Q3rsh_#1
180
140
100
75
60
45
35
25
15
10
← anti-HA antibody

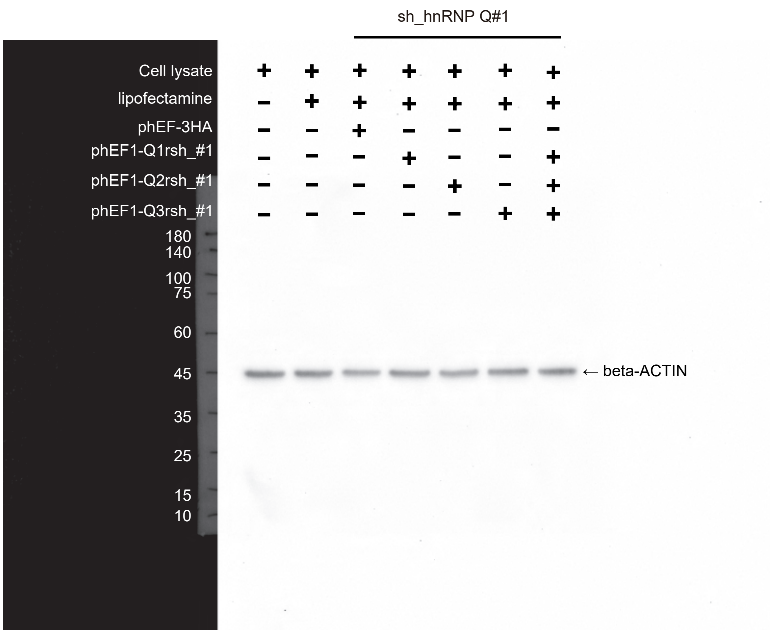
sh_hnRNP Q#1
Cell lysate
lipofectamine
phEF-3HA
phEF1-Q1rsh_#1
phEF1-Q2rsh_#1
phEF1-Q3rsh_#1
180
140
100
75
60
45
35
25
15
10
← beta-ACTIN

Figure 3A

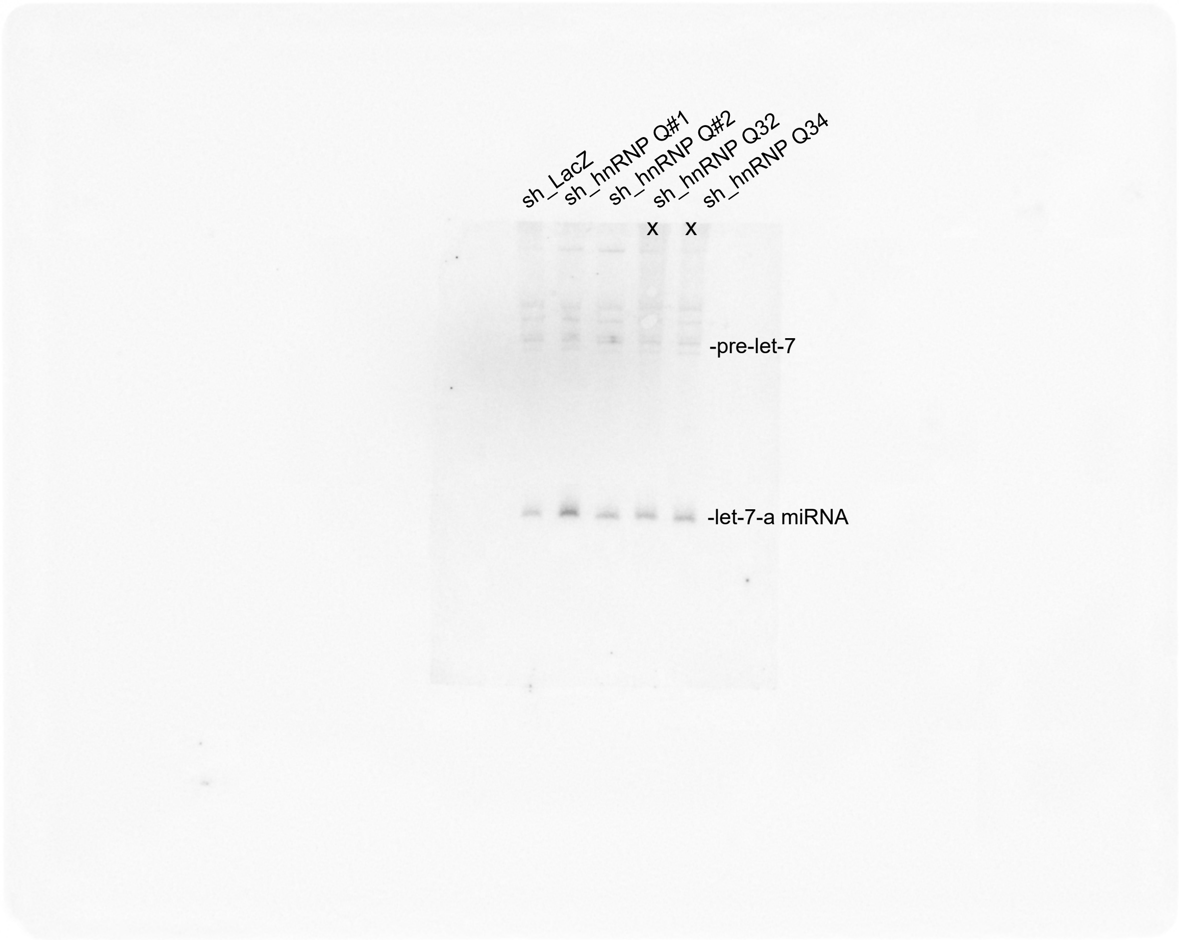
sh_LacZ
sh_hnRNP Q#1
sh_hnRNP Q#2
sh_hnRNP Q32
sh_hnRNP Q34
x x
-pre-let-7
-let-7-a miRNA

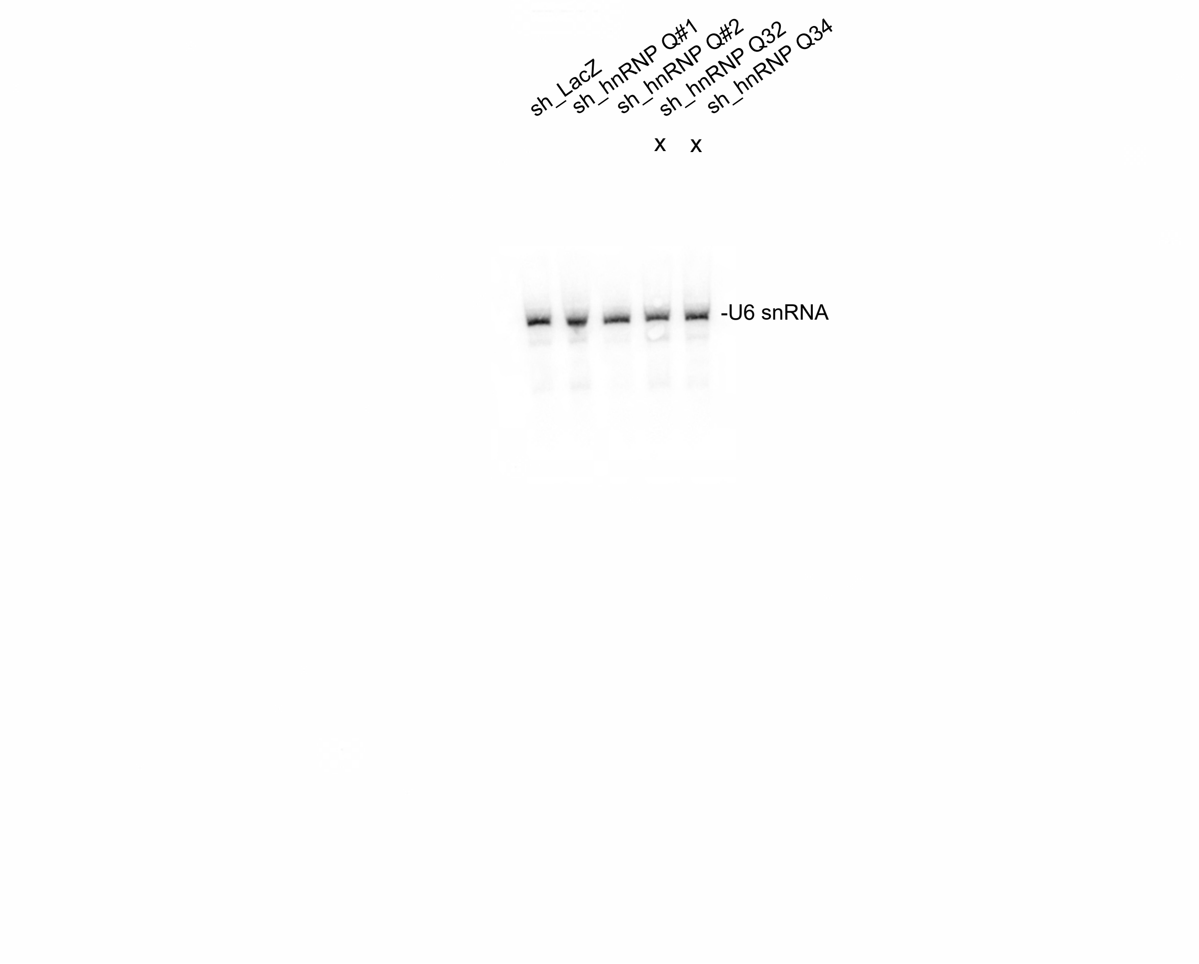
sh_LacZ
sh_hnRNP Q#1
sh_hnRNP Q#2
sh_hnRNP Q32
sh_hnRNP Q34
x x
-U6 snRNA

Figure 3C

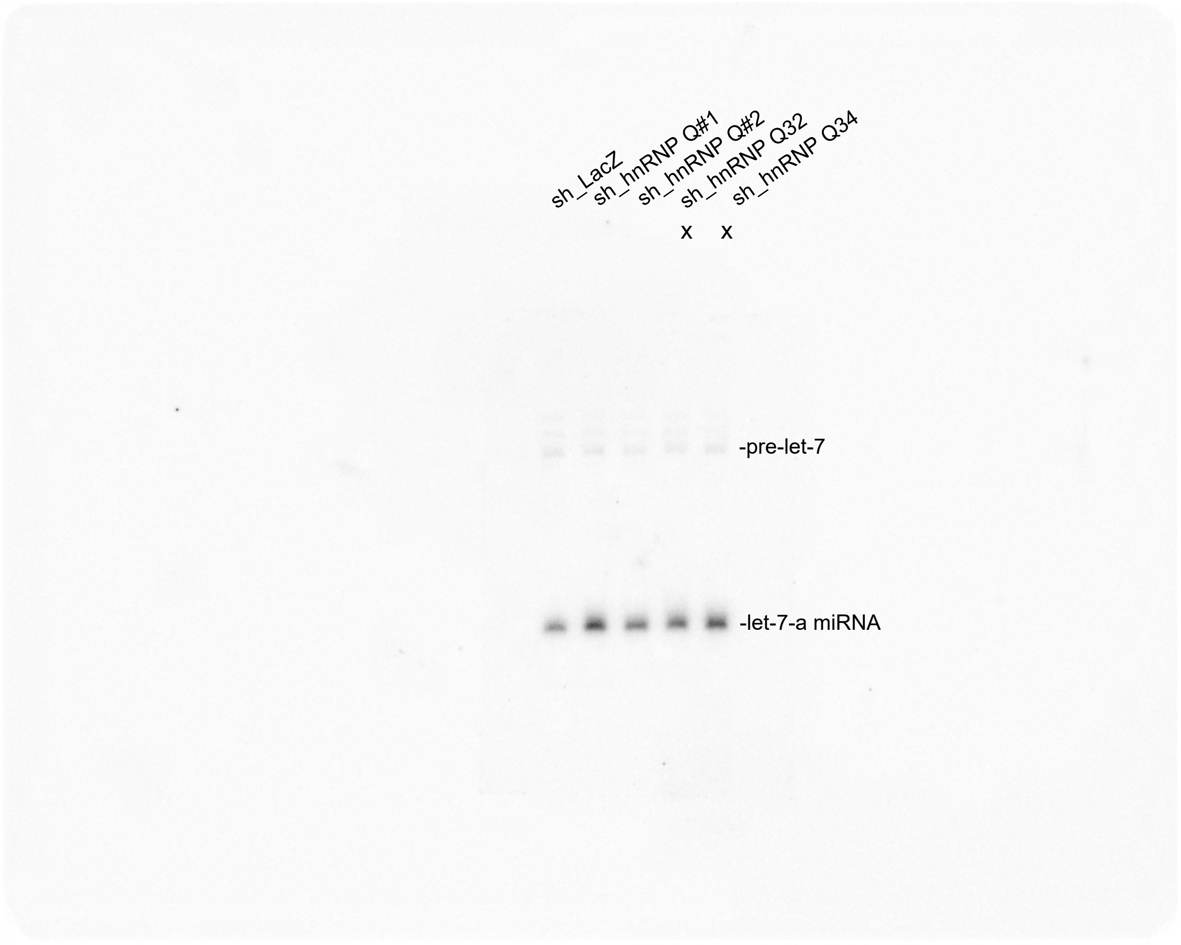
sh_LacZ
sh_hnRNP Q#1
sh_hnRNP Q#2
sh_hnRNP Q32
sh_hnRNP Q34
x x
-pre-let-7
-let-7-a miRNA

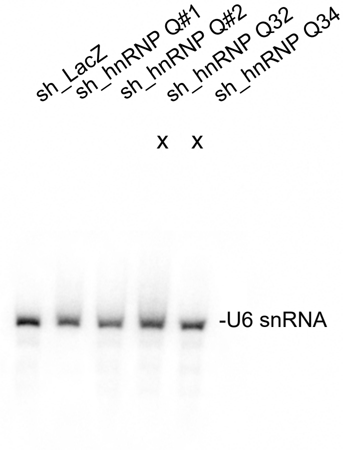
sh_LacZ
sh_hnRNP Q#1
sh_hnRNP Q#2
sh_hnRNP Q32
sh_hnRNP Q34
x x
-U6 snRNA

Figure 3E

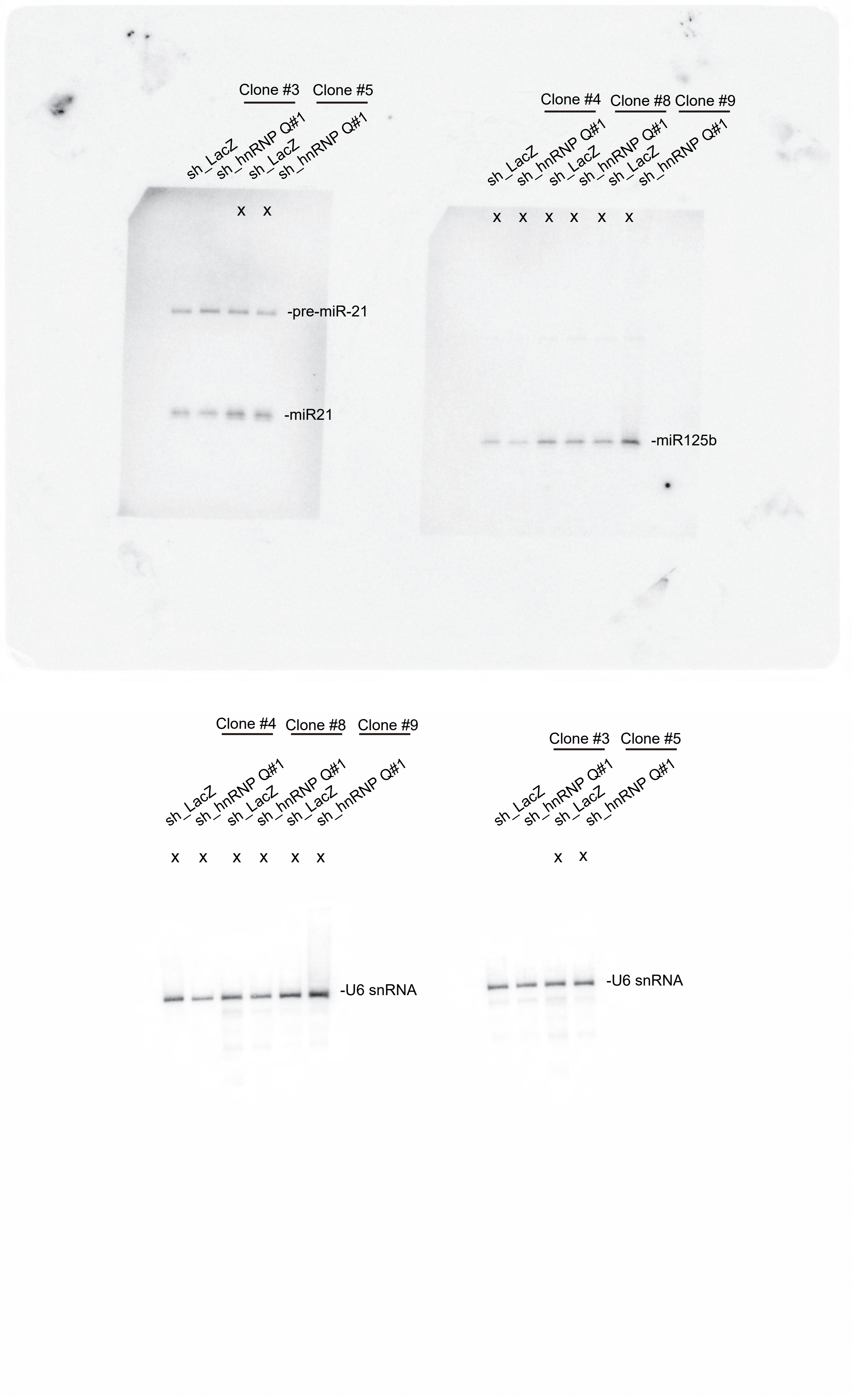

Clone #3
Clone #5
sh_LacZ
sh_hnRNP Q#1
sh_LacZ
sh_hnRNP Q#1
x x
-pre-miR-21
-miR21
Clone #4
Clone #8
Clone #9
sh_LacZ
sh_hnRNP Q#1
sh_LacZ
sh_hnRNP Q#1
sh_LacZ
sh_hnRNP Q#1
x x x x x x
-miR125b
Clone #4
Clone #8
Clone #9
sh_LacZ
sh_hnRNP Q#1
sh_LacZ
sh_hnRNP Q#1
sh_LacZ
sh_hnRNP Q#1
x x x x x x
-U6 snRNA
Clone #3
Clone #5
sh_LacZ
sh_hnRNP Q#1
sh_LacZ
sh_hnRNP Q#1
x x
-U6 snRNA

Figure 4B

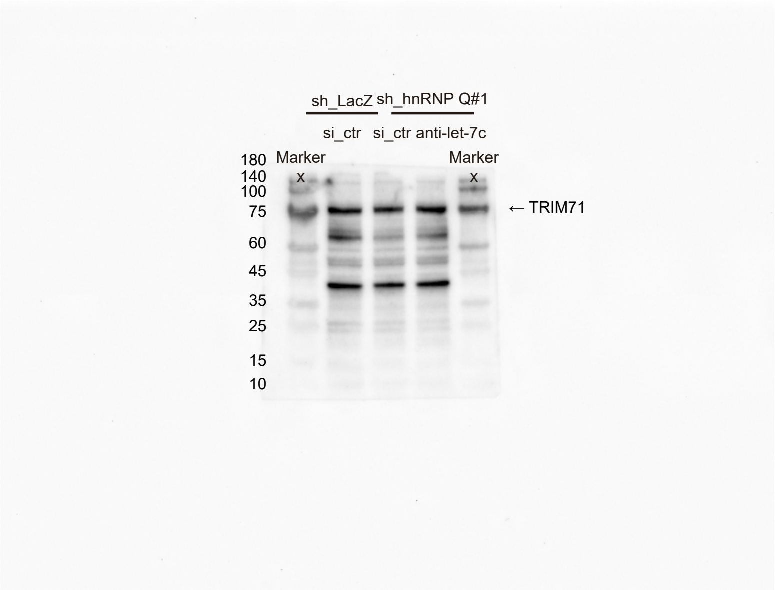

sh_LacZ sh_hnRNP Q#1
si_ctr si_ctr anti-let-7c
180 140 100 75 60 45 35 25 15 10
Marker x
Marker x
← TRIM71

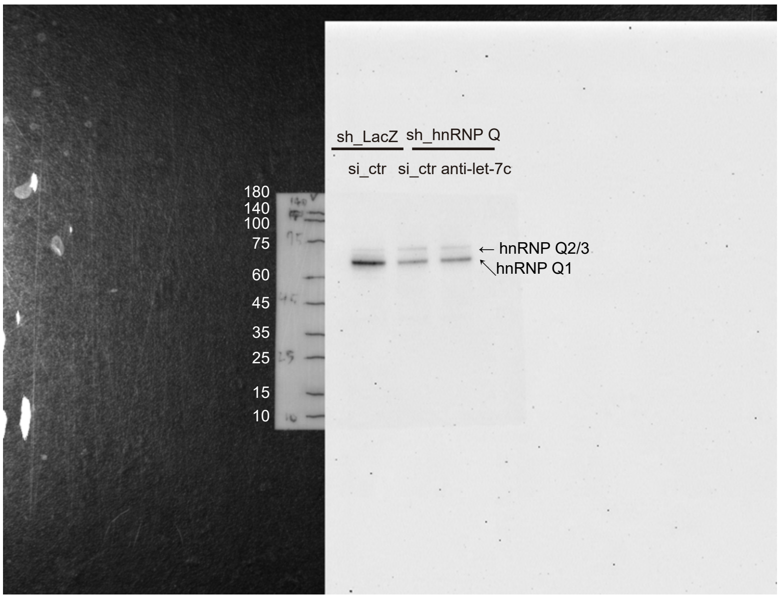

sh_LacZ sh_hnRNP Q
si_ctr si_ctr anti-let-7c
180 140 100 75 60 45 35 25 15 10
← hnRNP Q2/3
hnRNP Q1

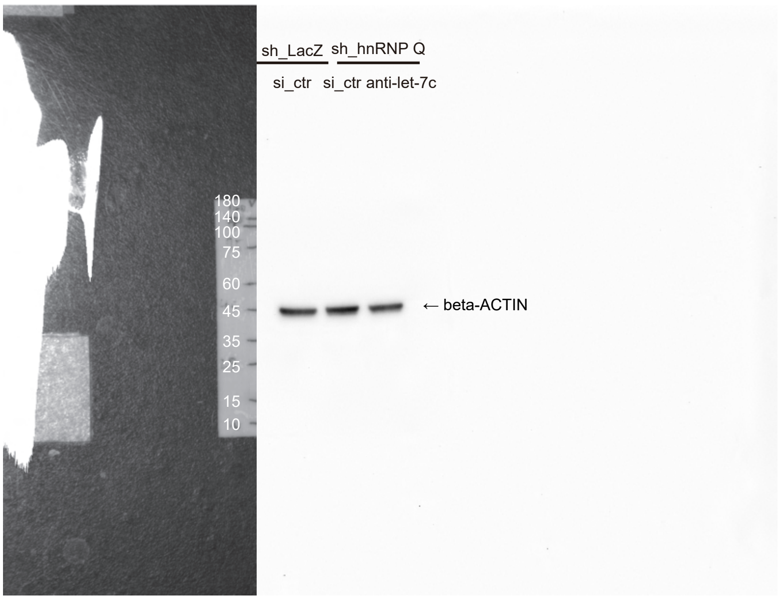

sh_LacZ sh_hnRNP Q
si_ctr si_ctr anti-let-7c
180 140 100 75 60 45 35 25 15 10
← beta-ACTIN

Supplementary Figure 2. (Bioreplicate #1)

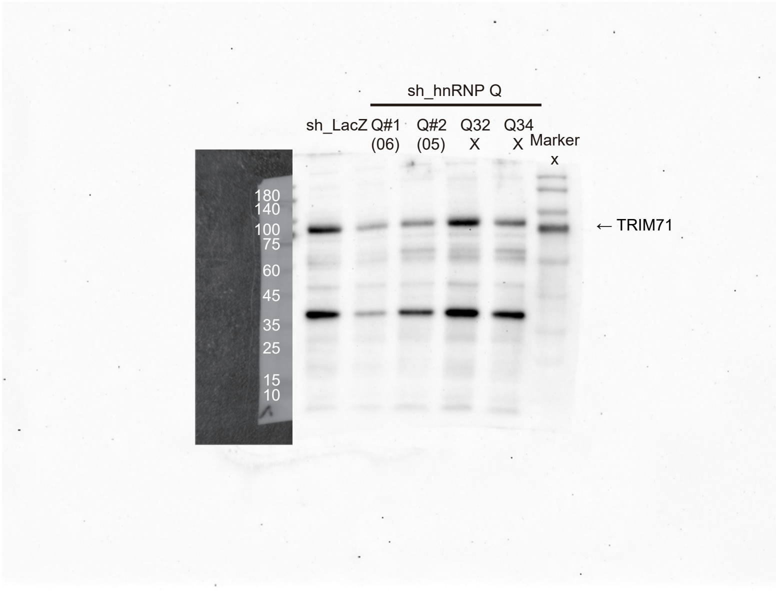

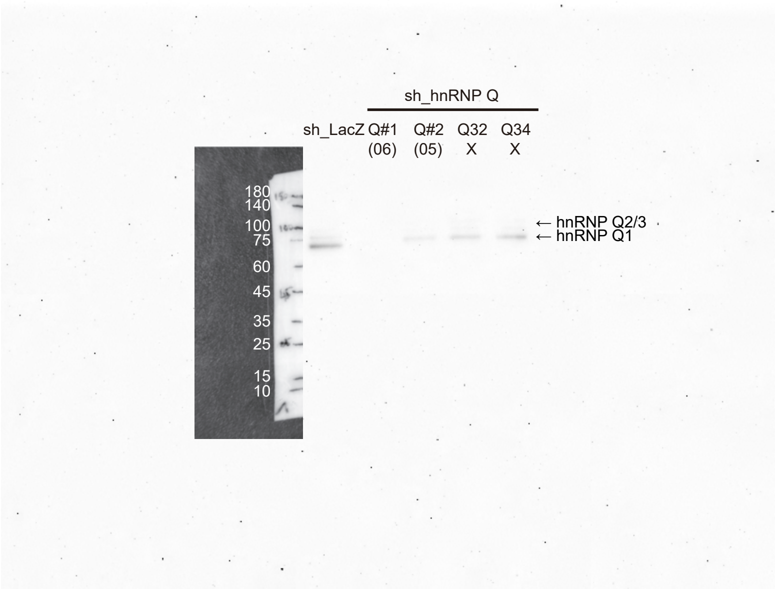

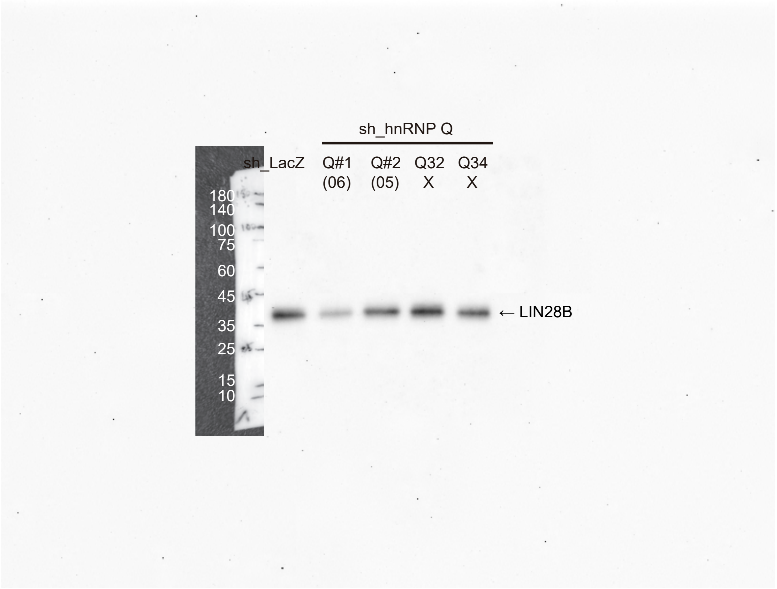

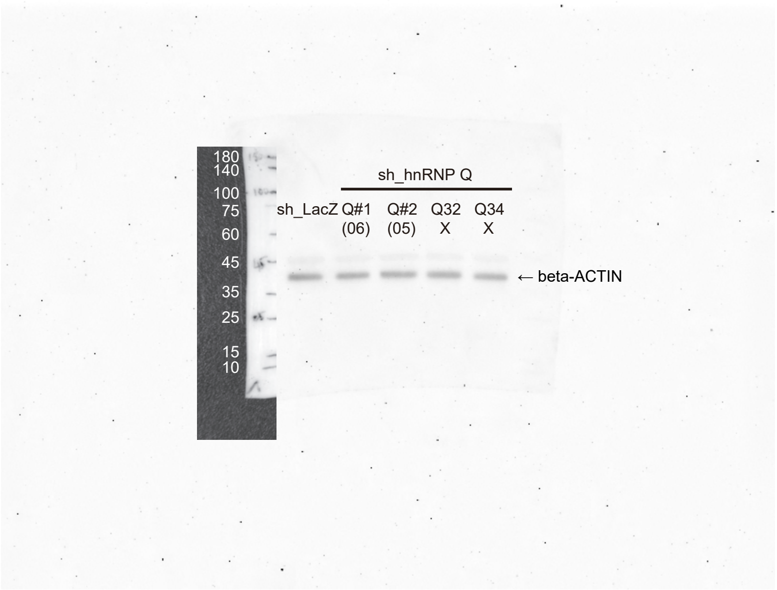

Supplementary Figure 2. (Bioreplicate #2)

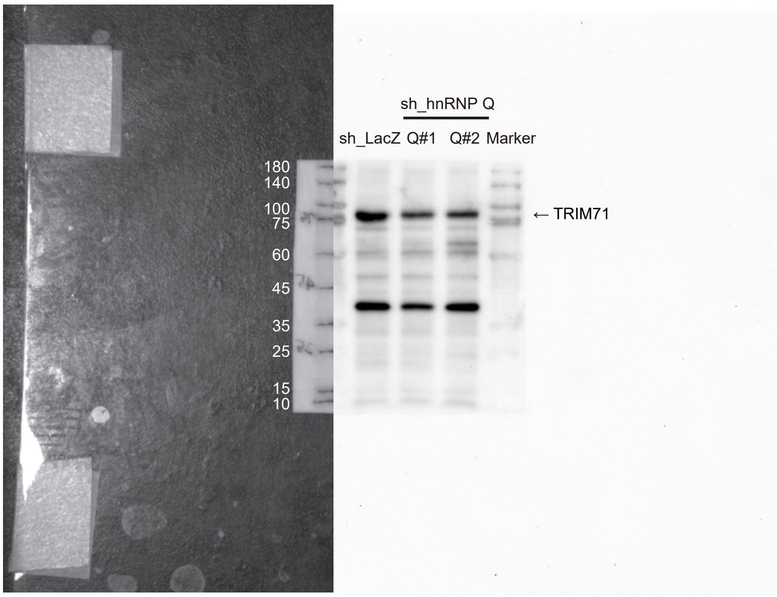

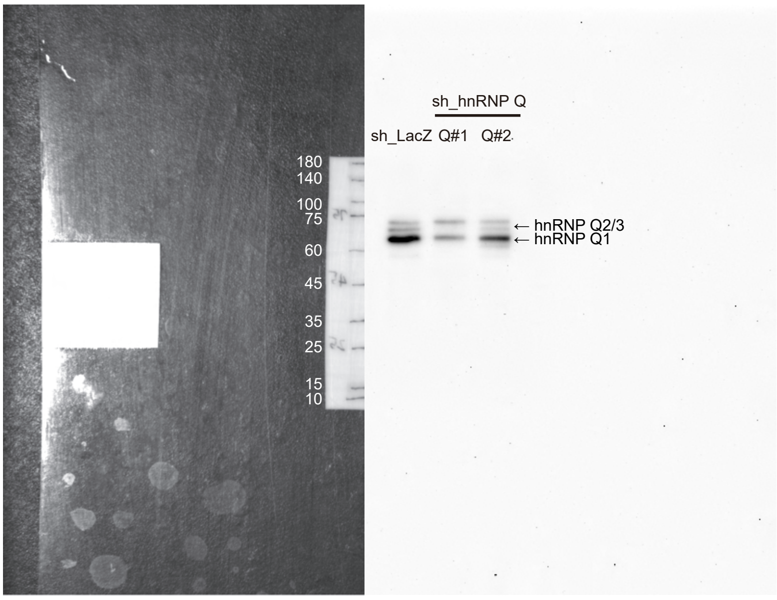

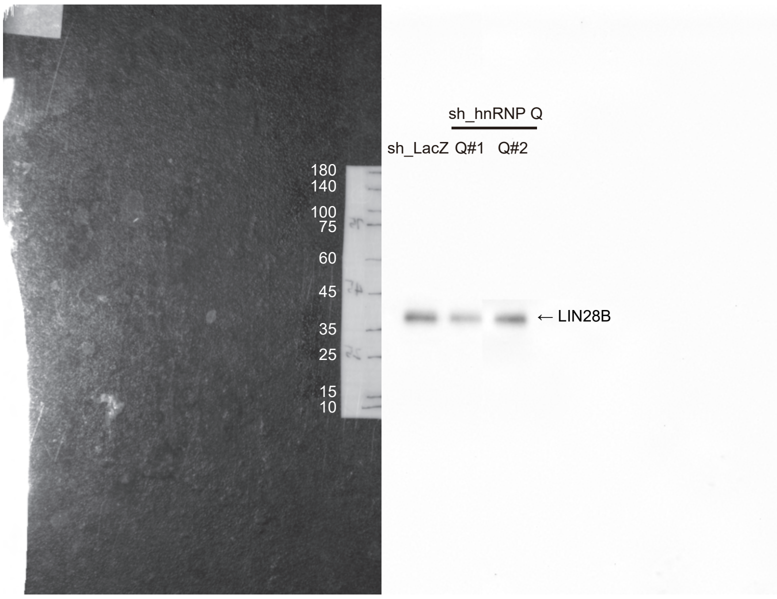

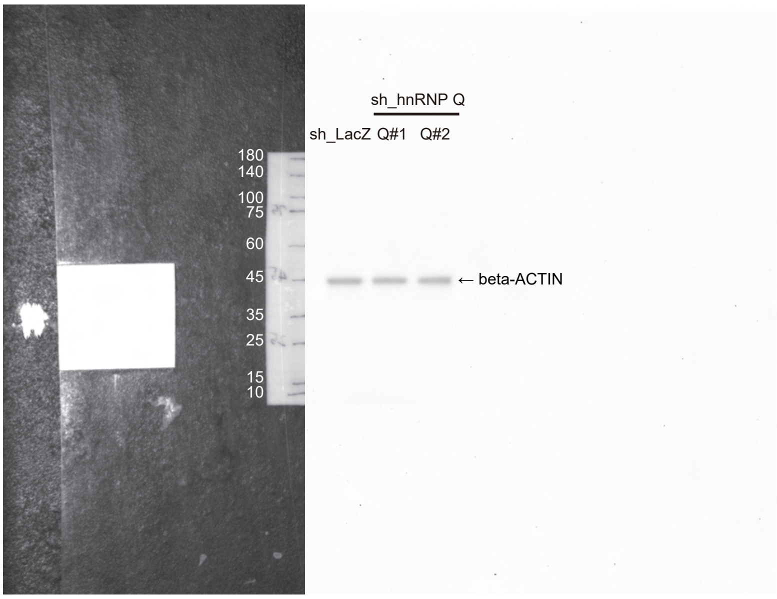

Supplementary Figure 2. (Bioreplicate #3)

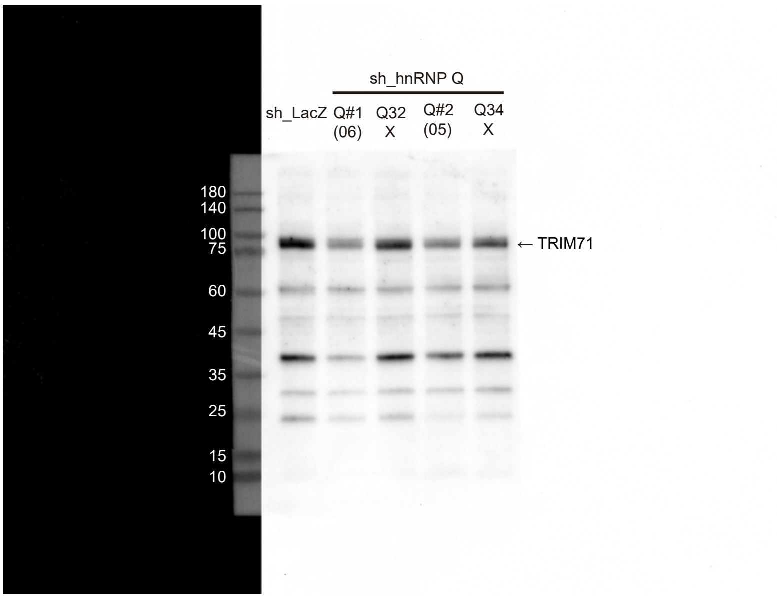

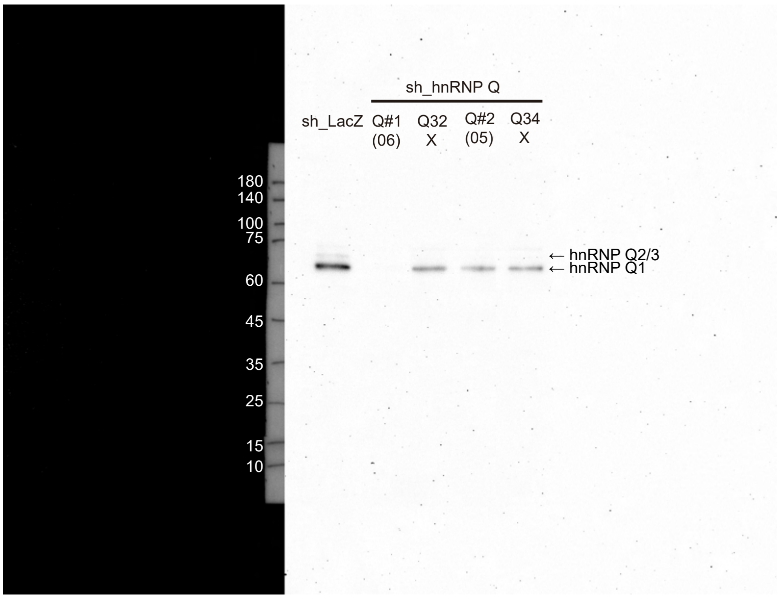

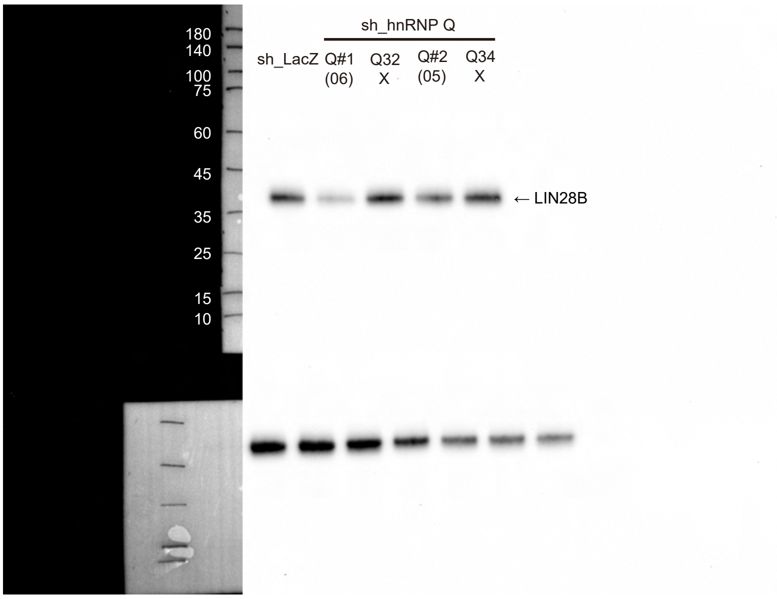

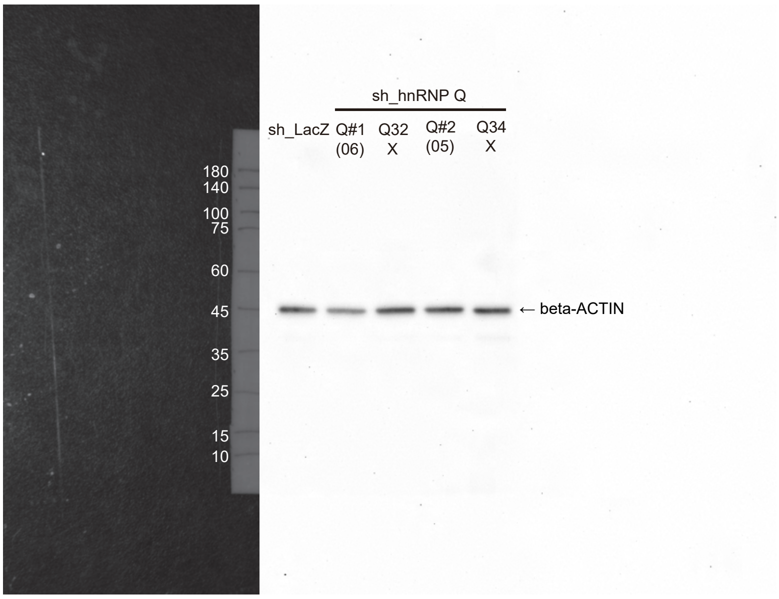

Supplementary Figure 3A (Bioreplicate #1)

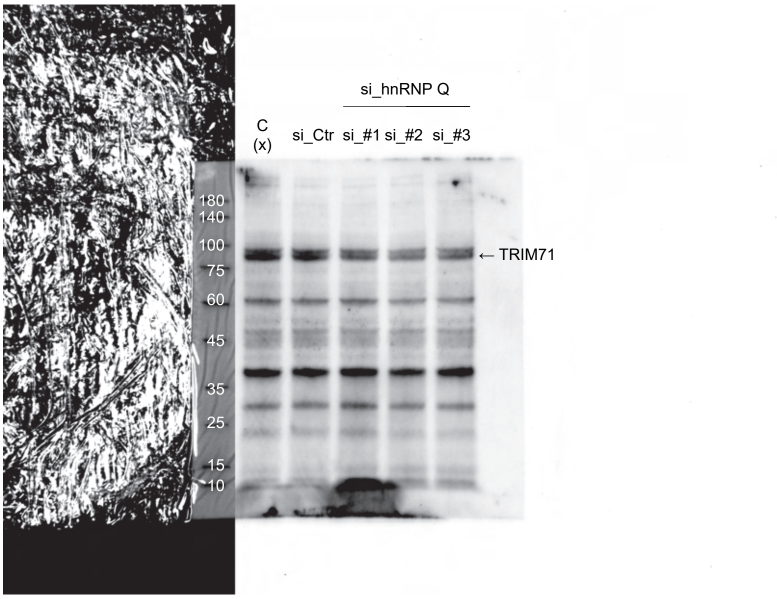

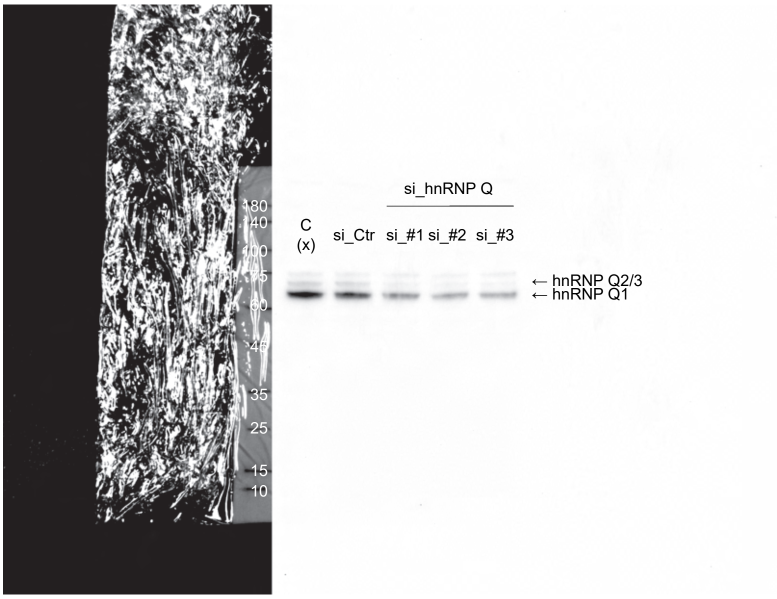

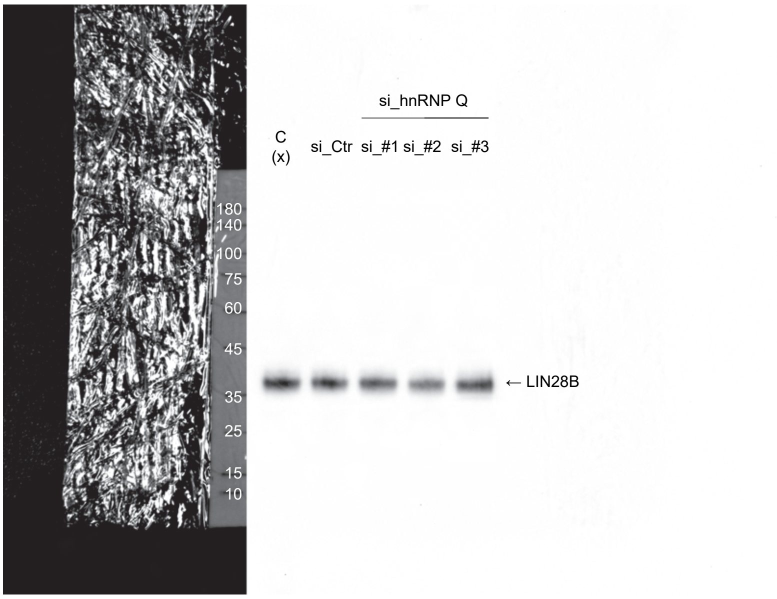

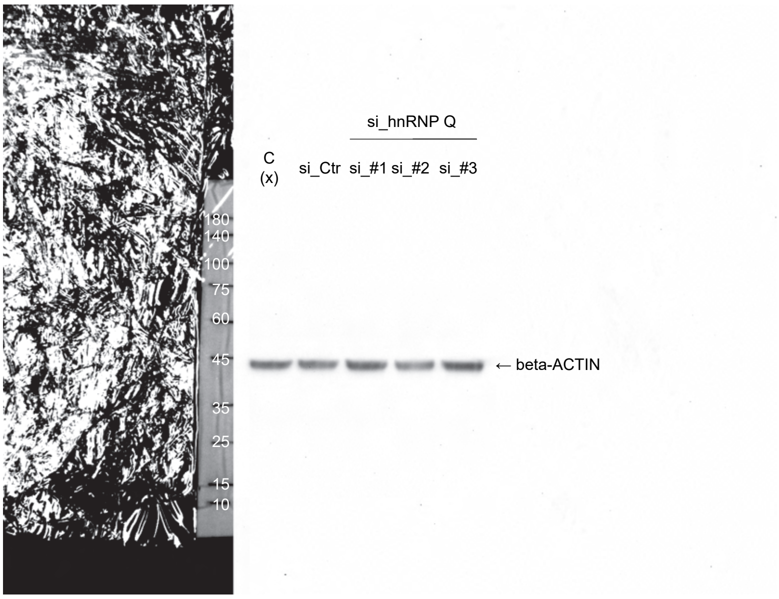

Supplementary Figure 3A (Bioreplicate #2)

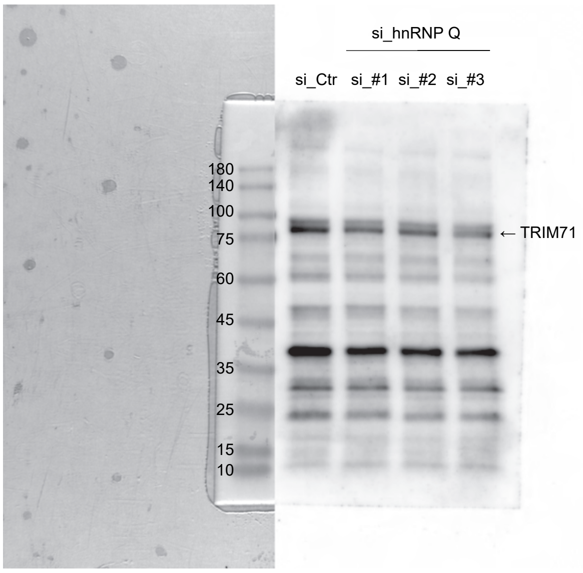

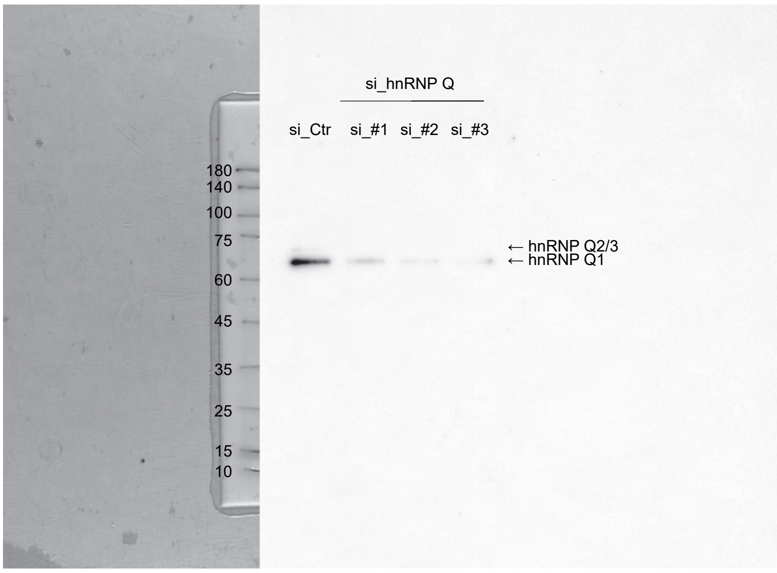

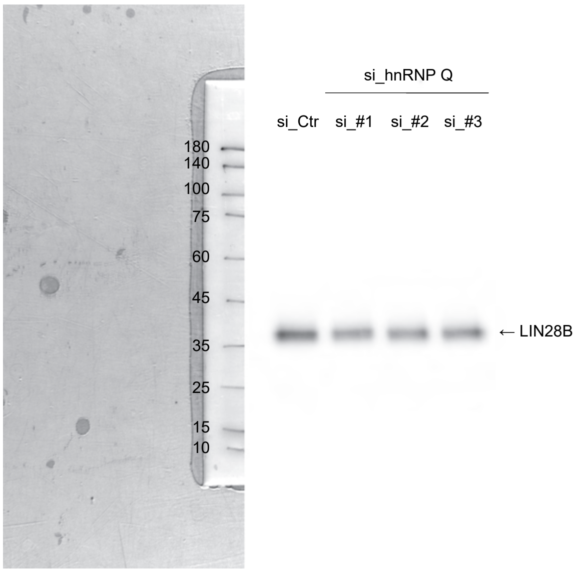

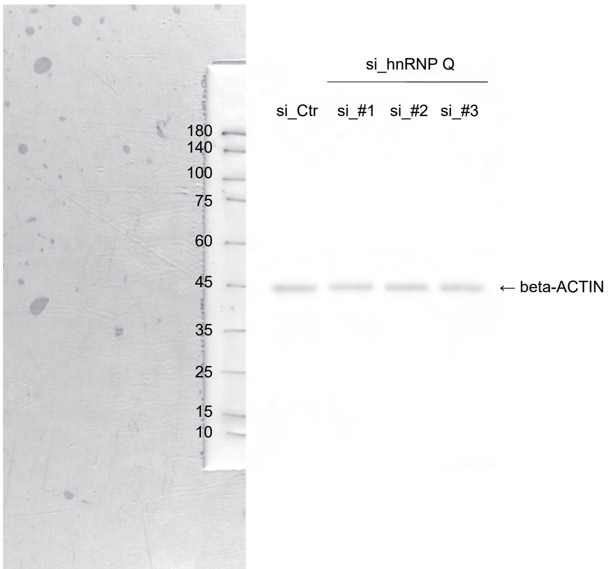

Supplementary Figure 3A (Bioreplicate #3)

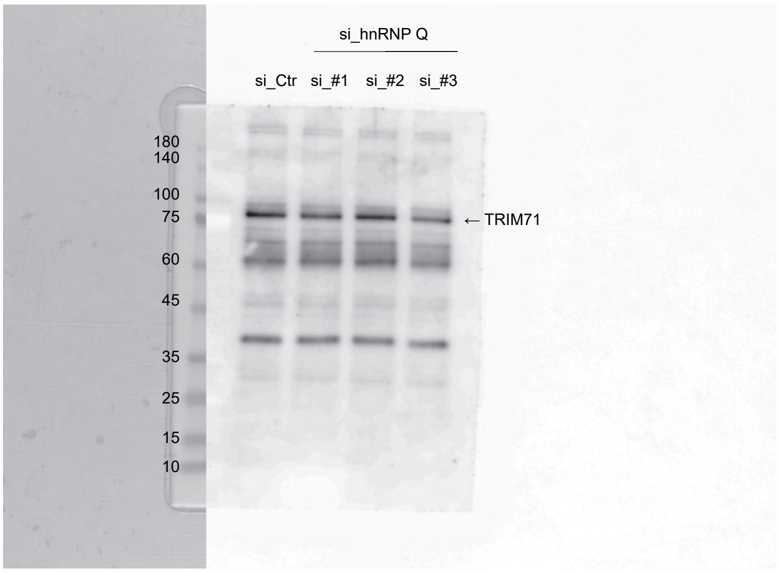

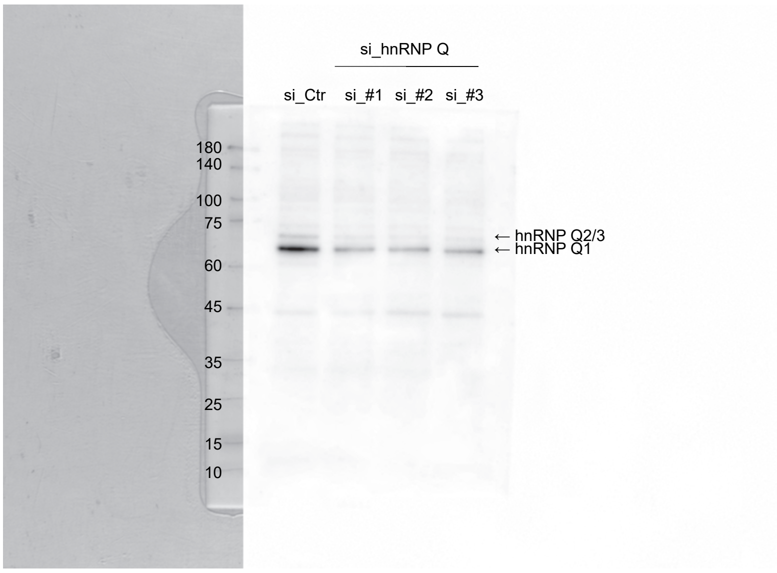

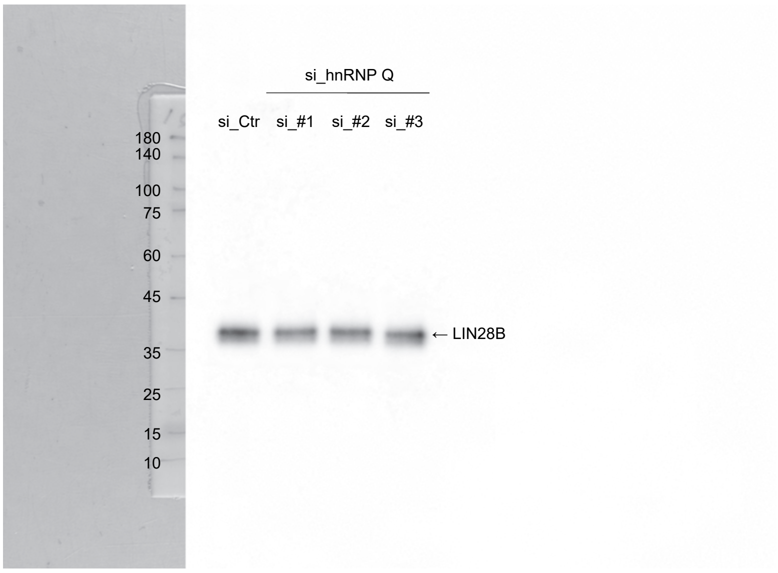

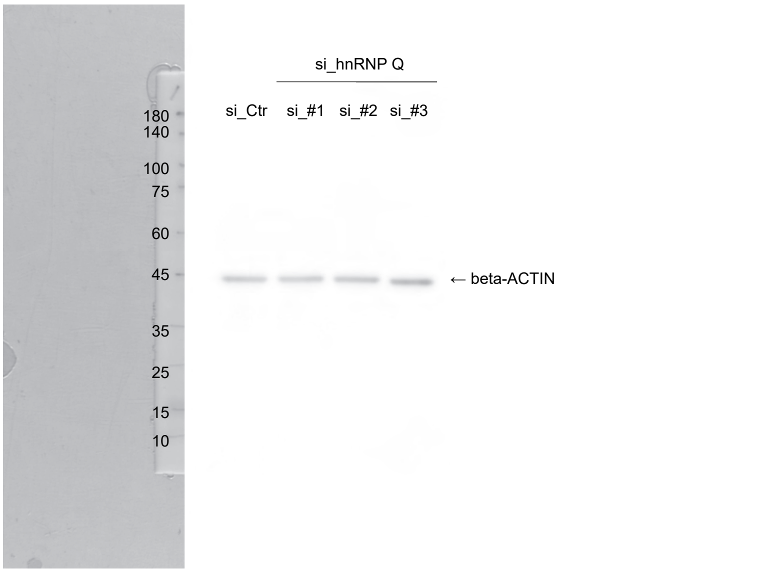

Supplementary Figure 4A

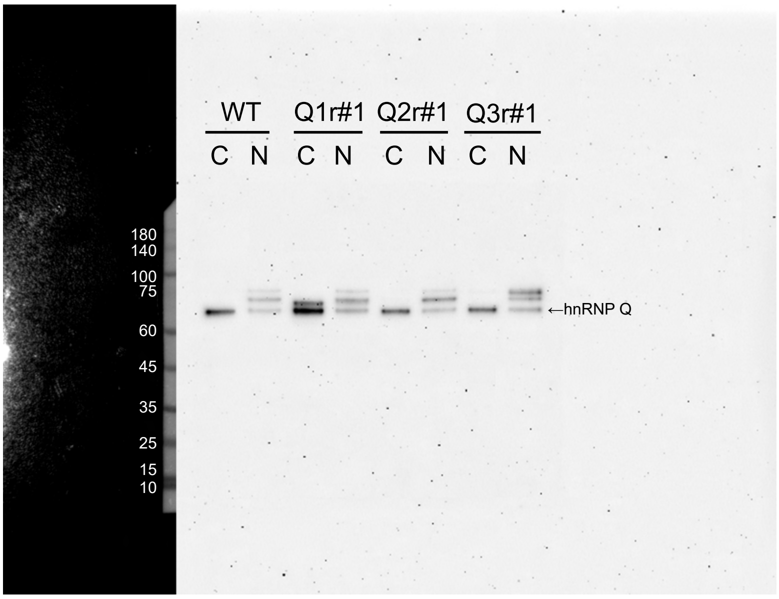

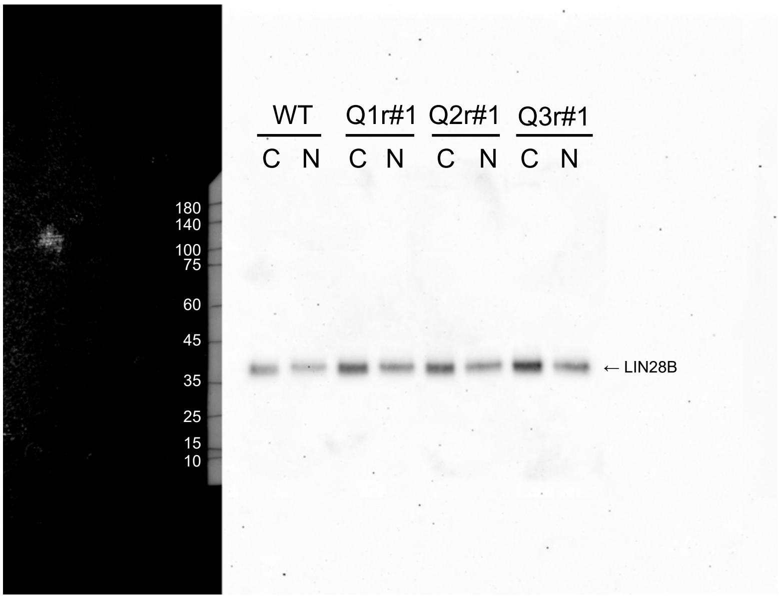

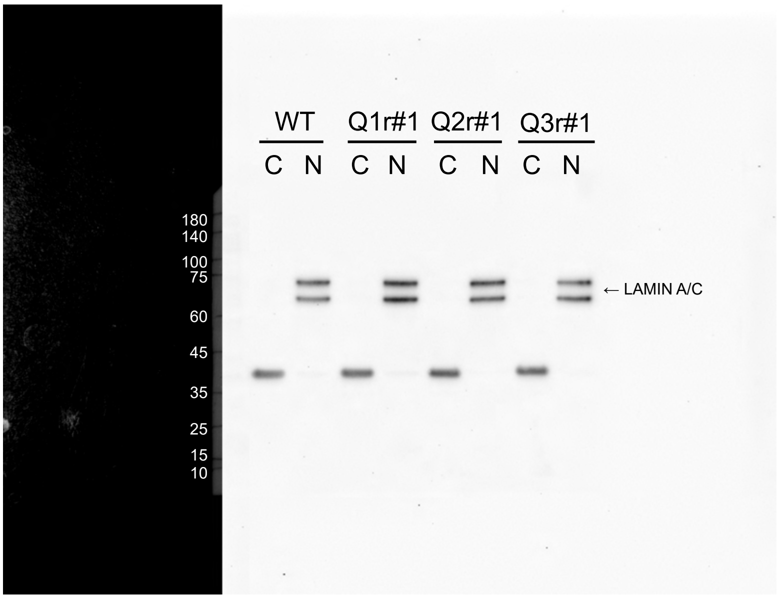

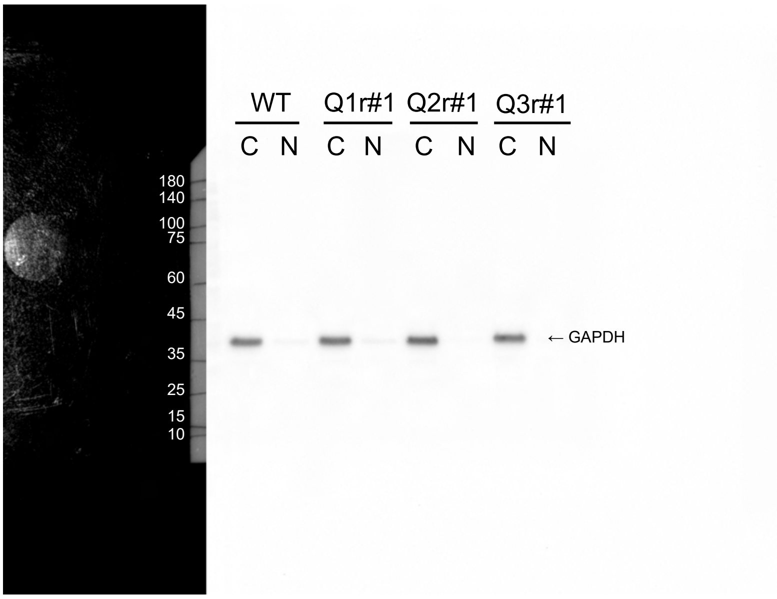

Supplementary Figure 4B

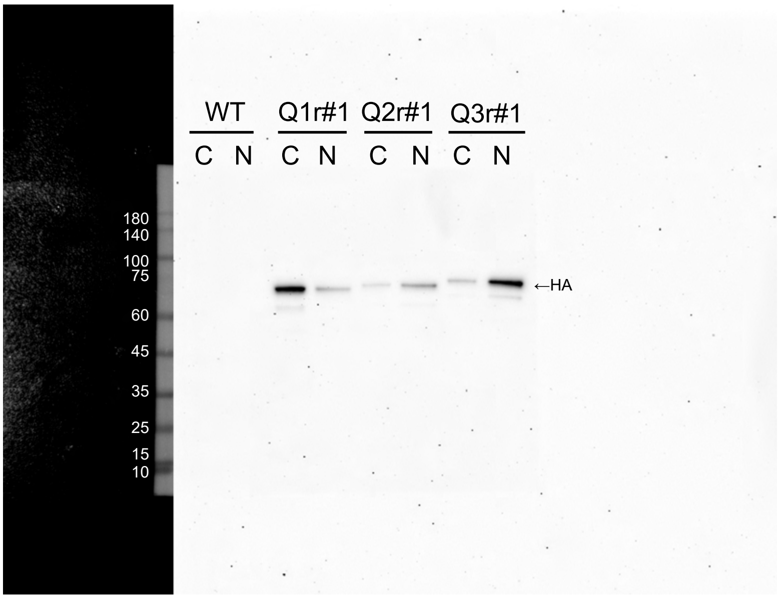

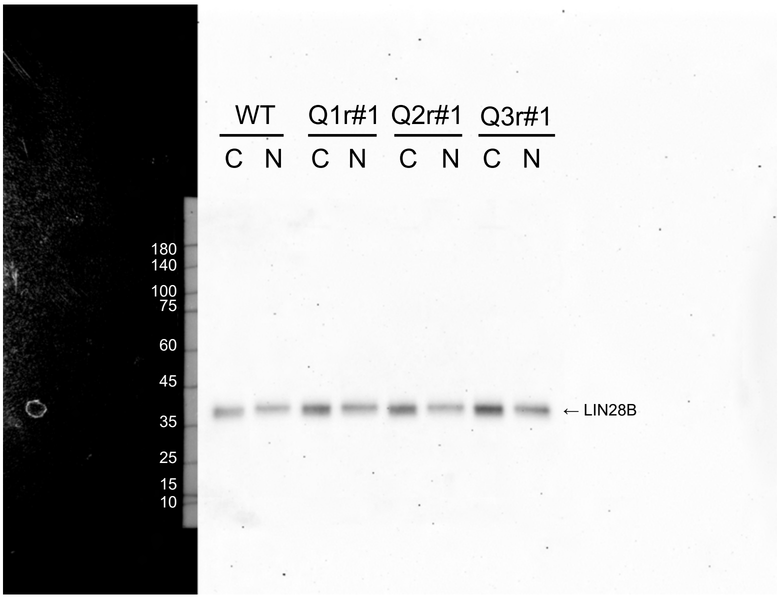

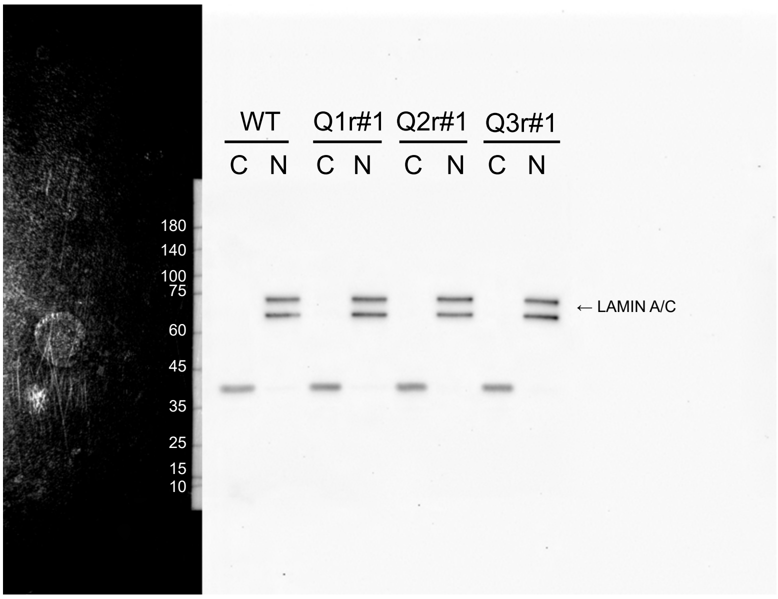

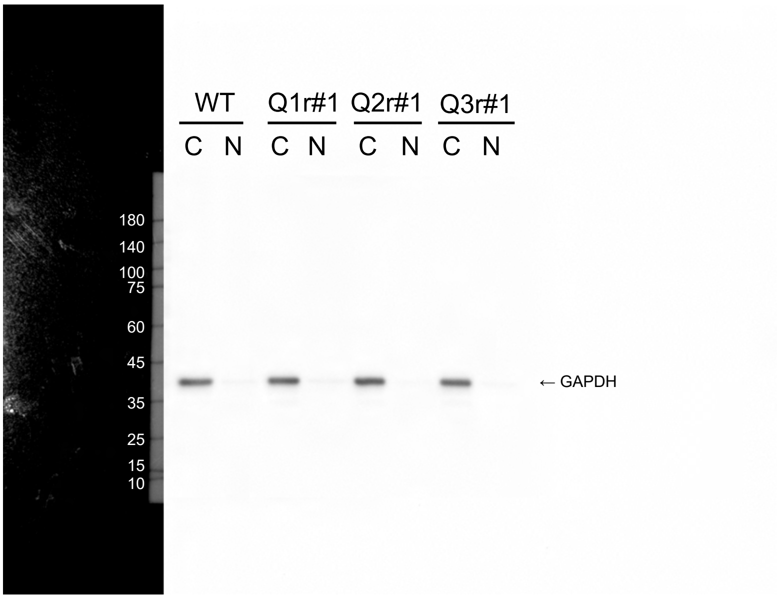

Supplementary Figure 5A (Bioreplicate #1)

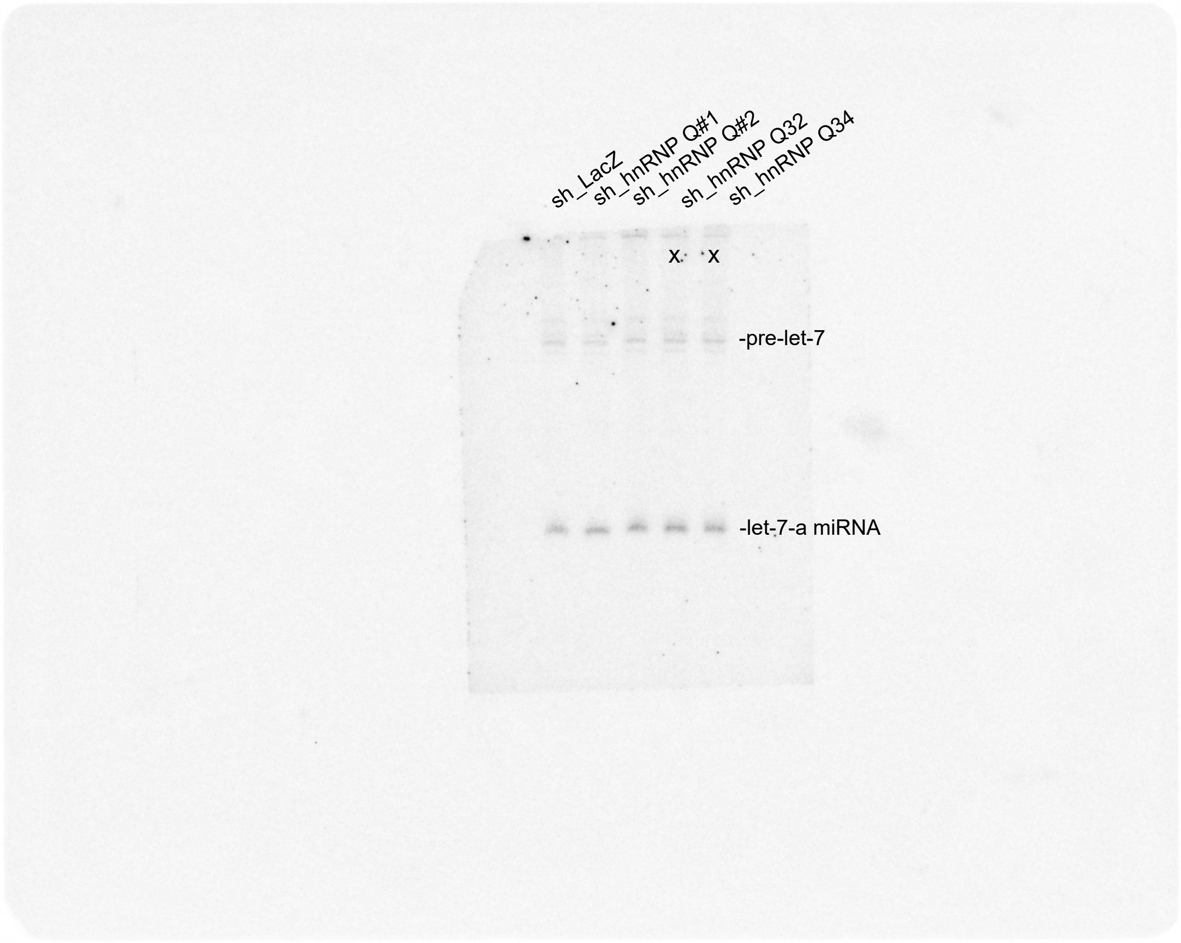

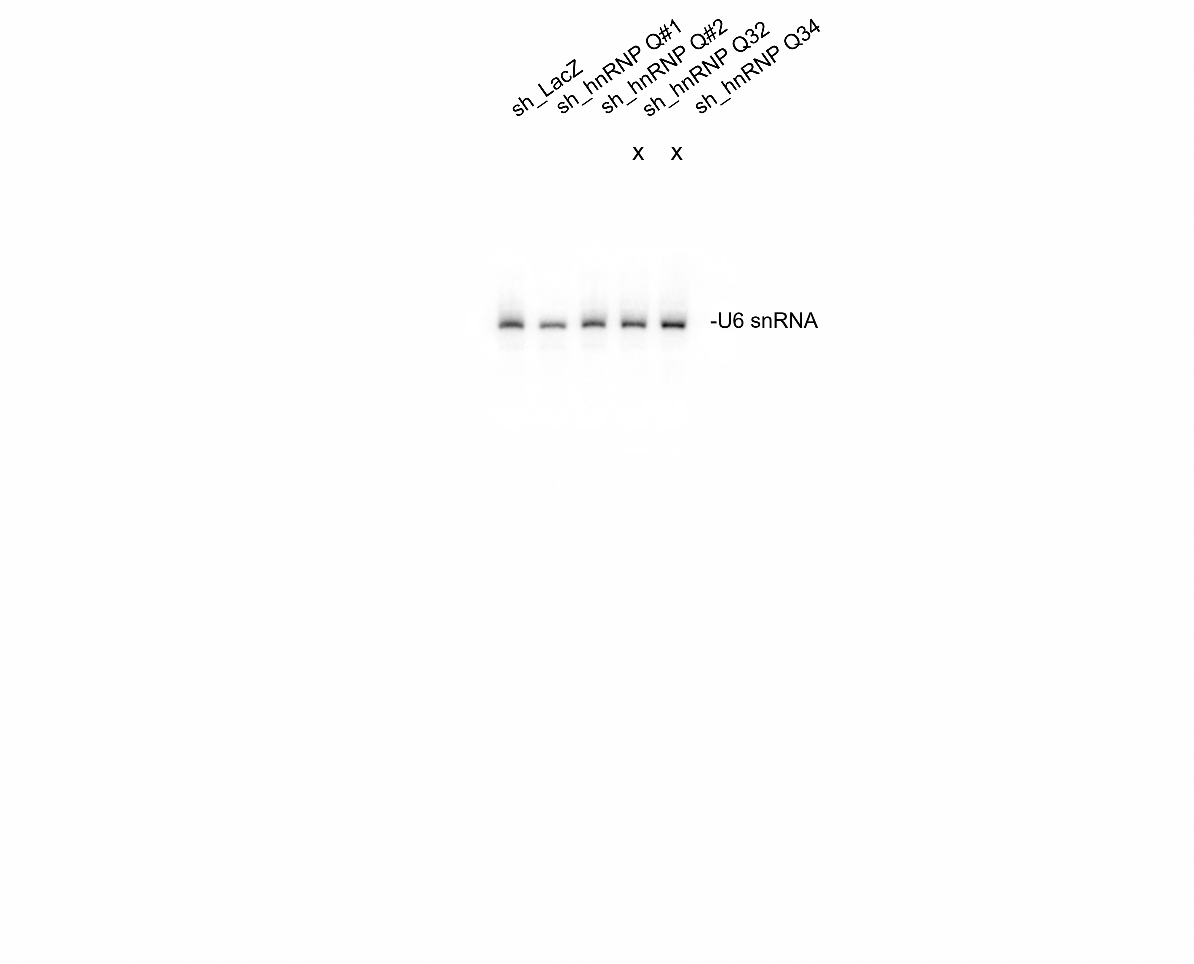

Supplementary Figure 5A (Bioreplicate #2)

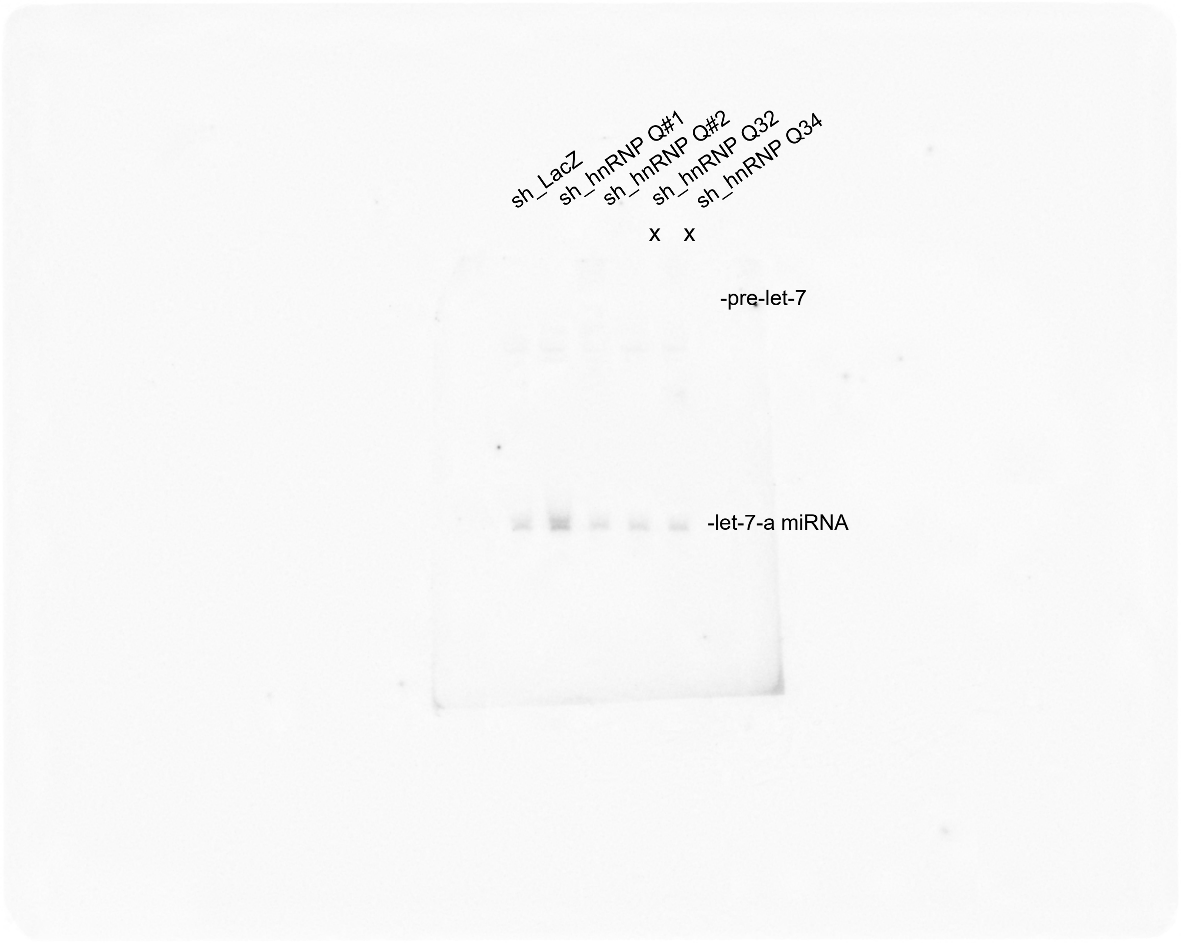

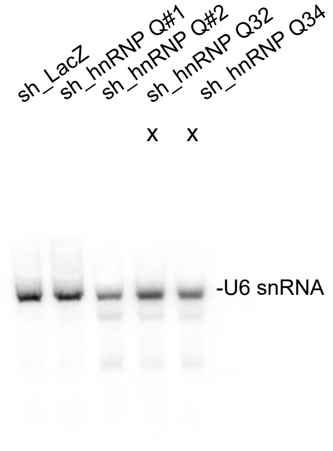

Supplementary Figure 5B (Bioreplicate #1)

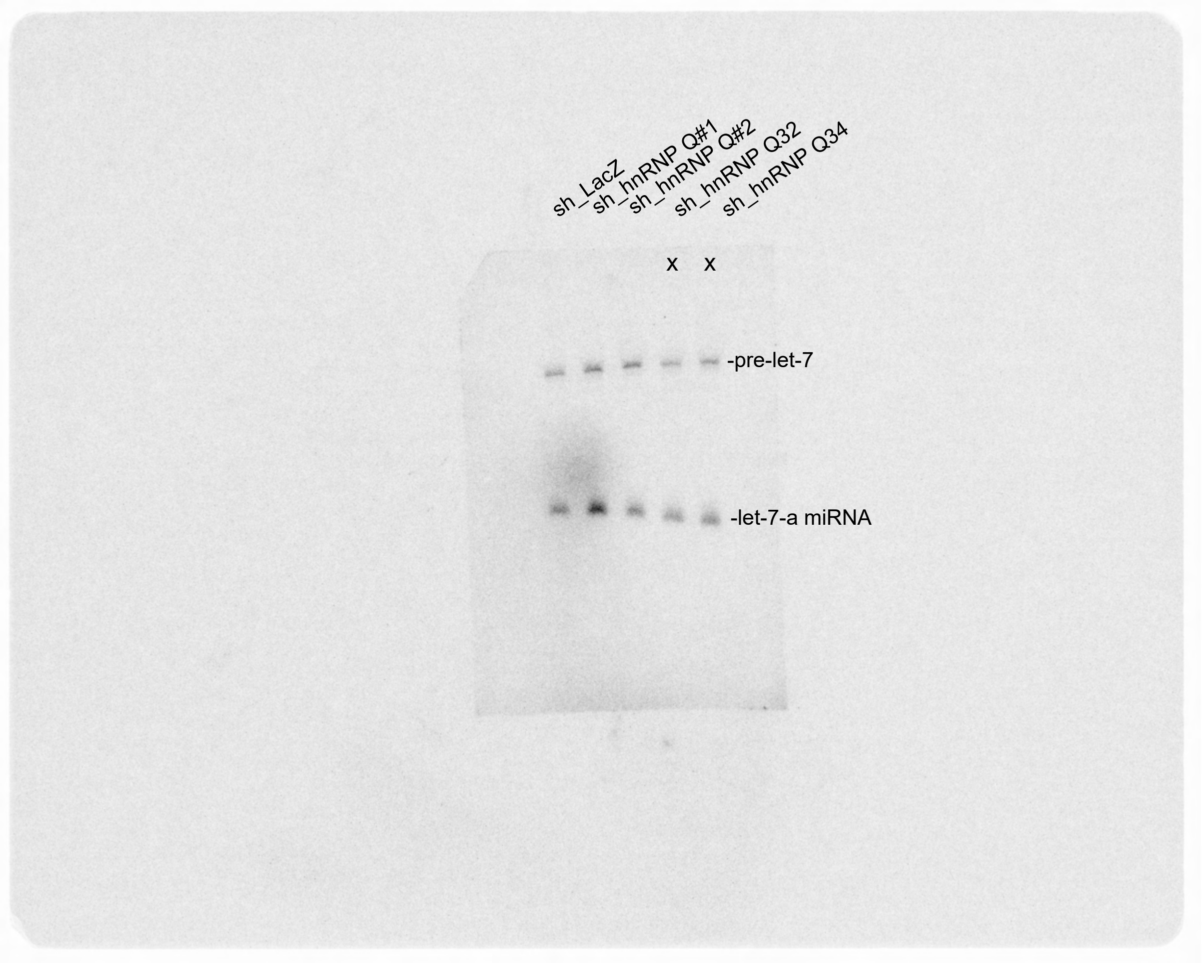

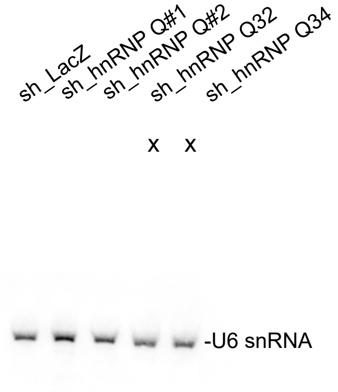

Supplementary Figure 5B (Bioreplicate #2)

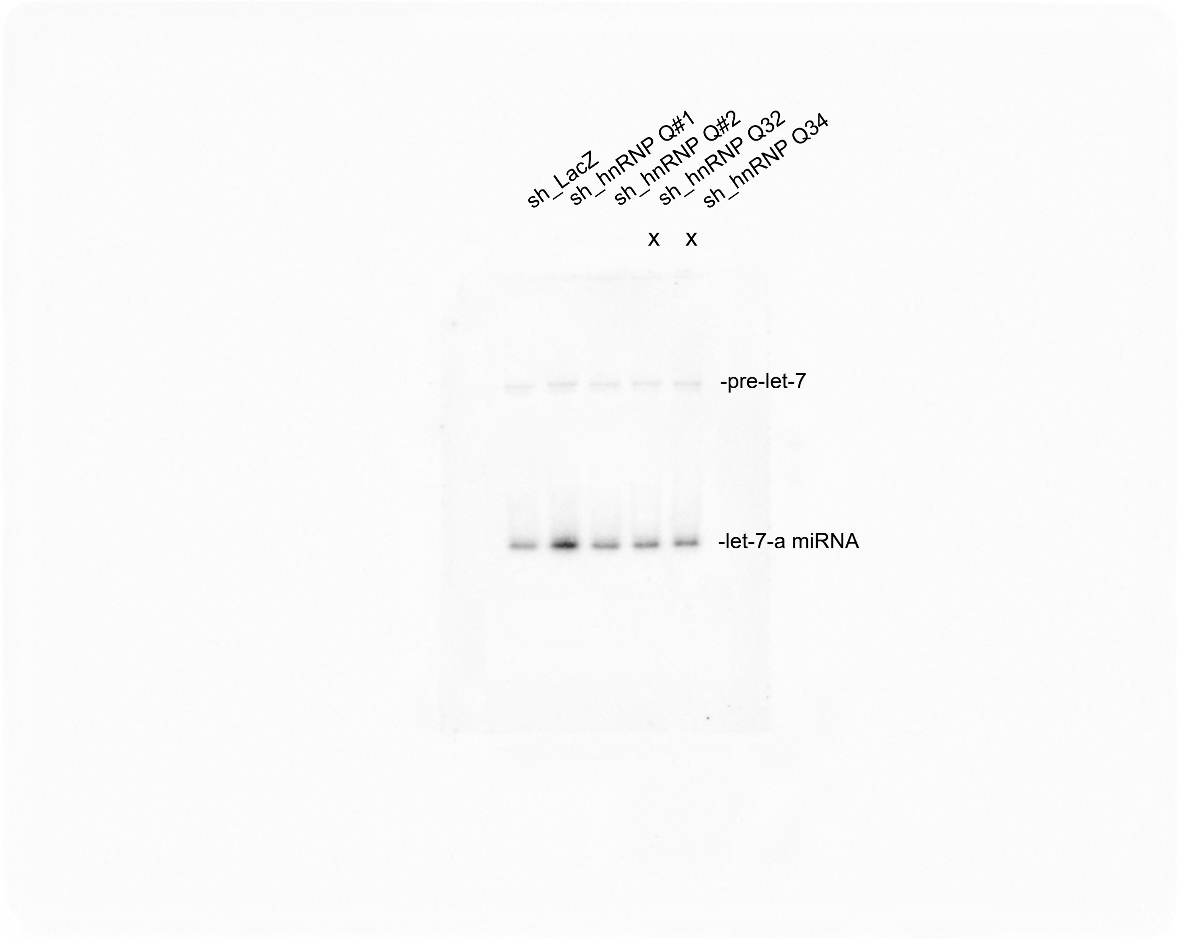

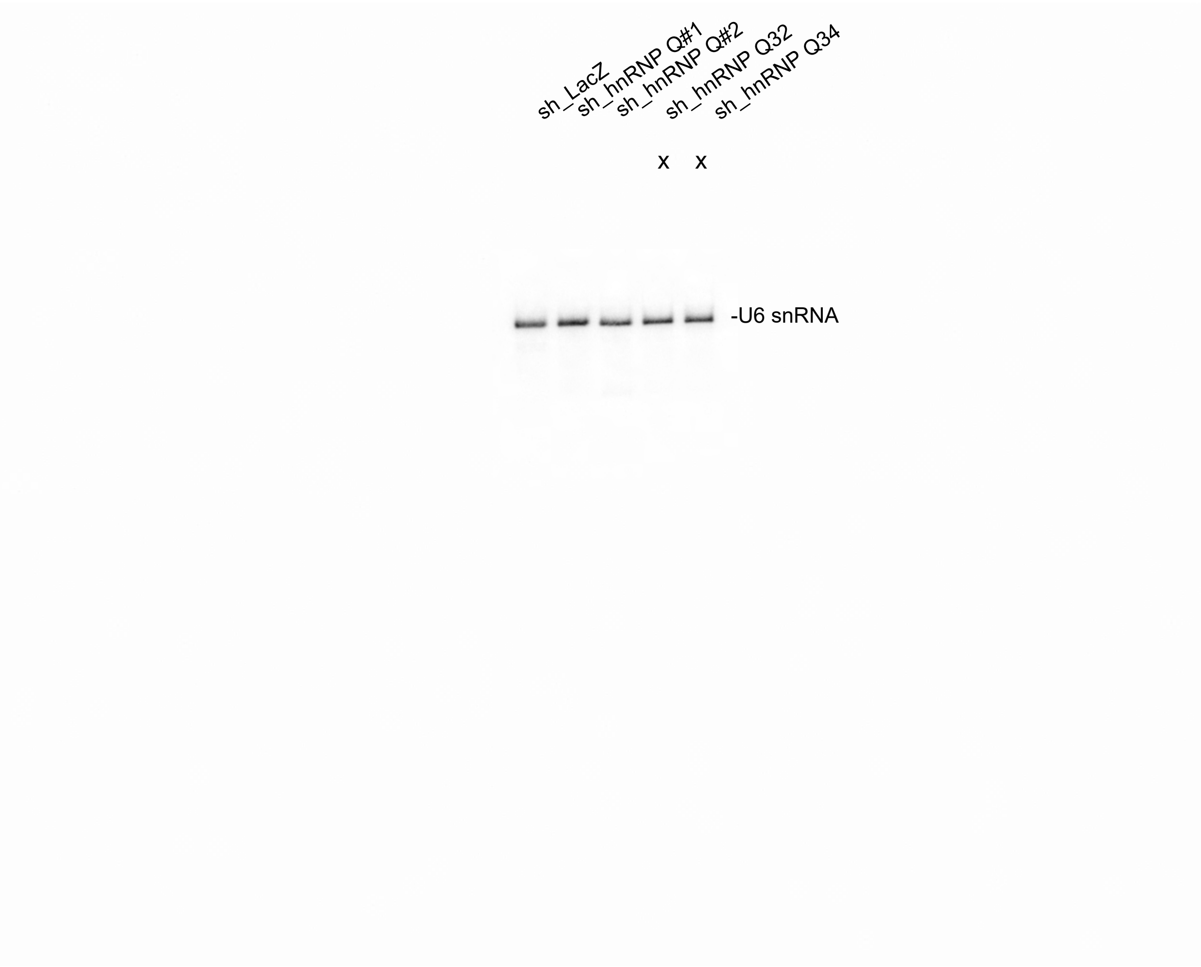

Supplementary Figure 5C

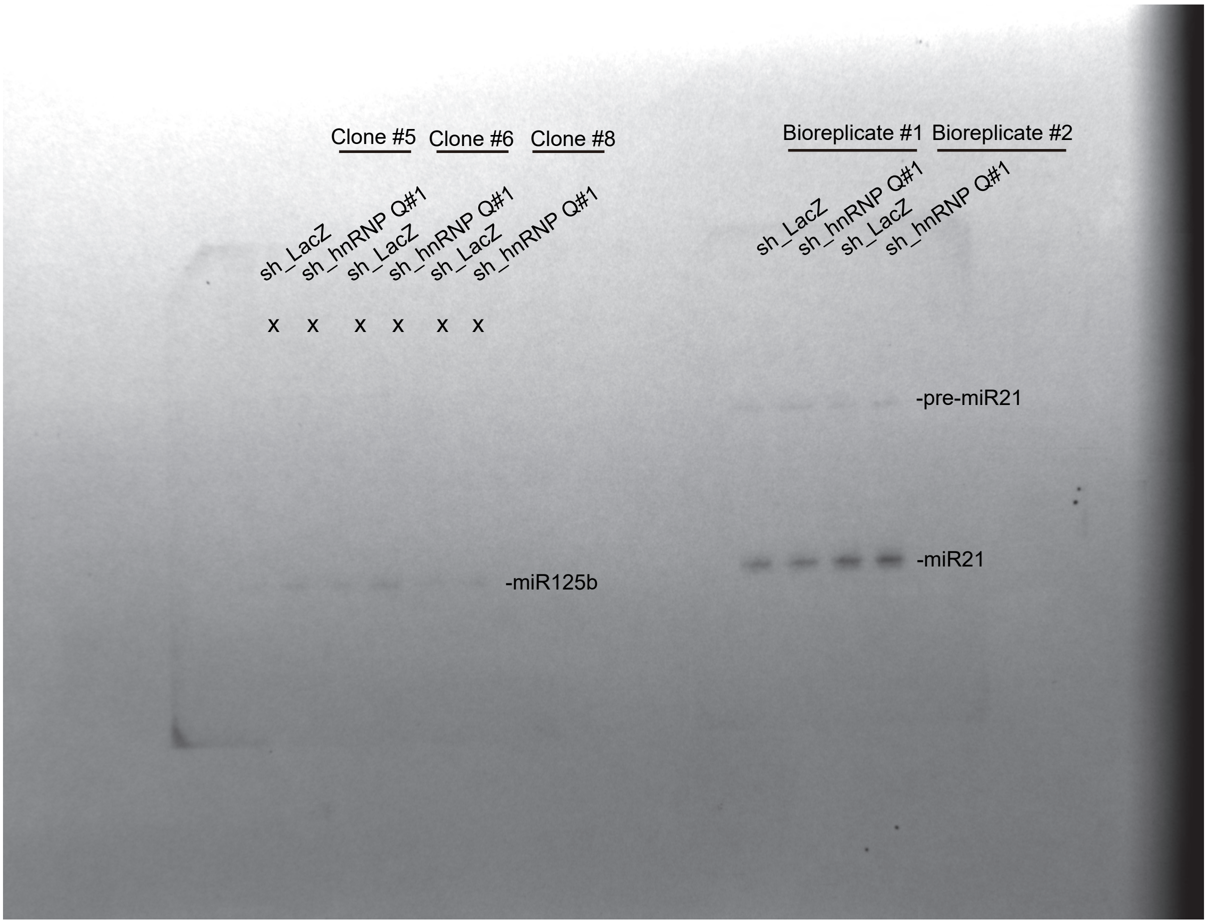

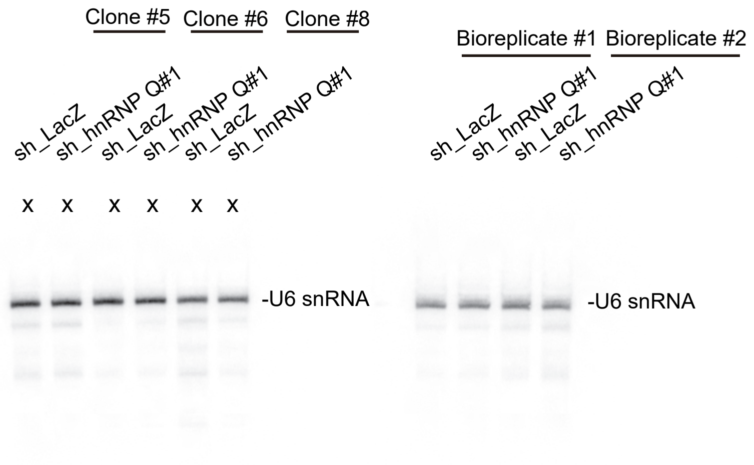

Supplement: S1 Raw images — (PDF) [file pone.0304947.s009.pdf]
